# ScaffoldGVAE: scaffold generation and hopping of drug molecules via a variational autoencoder based on multi-view graph neural networks

**DOI:** 10.1186/s13321-023-00766-0

**Published:** 2023-10-04

**Authors:** Chao Hu, Song Li, Chenxing Yang, Jun Chen, Yi Xiong, Guisheng Fan, Hao Liu, Liang Hong

**Affiliations:** 1 School of Physics and Astronomy and Institute of Natural Sciences, Shanghai Jiao Tong University, Shanghai, 200240 China; 2 Shanghai Matwings Technology Co., Ltd., Shanghai, 200240 China; 3 School of Information Science and Engineering, East China University of Science and Technology, Shanghai, 200237 China; 4 School of Life Sciences and Biotechnology, Shanghai Jiao Tong University, Shanghai, 200240 China; 5 Zhangjiang Institute for Advanced Study, Shanghai Jiao Tong University, Shanghai, 201203 China

**Keywords:** Drug design, Molecule generation, Scaffold hopping, Variational autoencoder, Multi-view graph neural networks

## Abstract

In recent years, drug design has been revolutionized by the application of deep learning techniques, and molecule generation is a crucial aspect of this transformation. However, most of the current deep learning approaches do not explicitly consider and apply scaffold hopping strategy when performing molecular generation. In this work, we propose ScaffoldGVAE, a variational autoencoder based on multi-view graph neural networks, for scaffold generation and scaffold hopping of drug molecules. The model integrates several important components, such as node-central and edge-central message passing, side-chain embedding, and Gaussian mixture distribution of scaffolds. To assess the efficacy of our model, we conduct a comprehensive evaluation and comparison with baseline models based on seven general generative model evaluation metrics and four scaffold hopping generative model evaluation metrics. The results demonstrate that ScaffoldGVAE can explore the unseen chemical space and generate novel molecules distinct from known compounds. Especially, the scaffold hopped molecules generated by our model are validated by the evaluation of GraphDTA, LeDock, and MM/GBSA. The case study of generating inhibitors of LRRK2 for the treatment of PD further demonstrates the effectiveness of ScaffoldGVAE in generating novel compounds through scaffold hopping. This novel approach can also be applied to other protein targets of various diseases, thereby contributing to the future development of new drugs. Source codes and data are available at https://github.com/ecust-hc/ScaffoldGVAE.

**Supplementary Information:**

The online version contains supplementary material available at 10.1186/s13321-023-00766-0.

## Introduction

The process of drug discovery is a complex and resource-intensive endeavor, involving significant human effort, material resources, and financial investment. One of the major challenges in drug discovery is the vast and discrete nature of the chemical space. However, recent advancements in artificial intelligence and the utilization of big data have begun to reshape this landscape. Deep learning approaches have emerged as powerful alternatives to traditional brute force methods like high-throughput screening. In particular, generative models have gained significant attention and have been applied to the design of de novo drug molecules, enabling the generation of new molecules with desired properties. Several drug molecular generation methods based on generative models have been developed in recent years. These include variations of the variational autoencoder (VAE) such as JT-VAE [1], GVAE [2], GraphVAE [3], NEVAE [4], and others. Additionally, there are generation methods based on the generative adversarial network (GAN) model, such as MolGAN [5], and ORGANIC [6]. RNN-based methods like MolRNN [7] and MolecularRNN [8], as well as diffusion model methods like GEOLDM [9], and MolDiff [10], have also been explored.

Scaffold hopping [11] is a widely employed strategy in drug design for traditional medicinal chemists, and when combined with artificial intelligence, it becomes a powerful tool for molecular optimization and drug design. The scaffold of a molecule plays a crucial role in determining its binding mode and interaction within the pocket of the protein. By modifying and optimizing the scaffold structure, we can discover more effective and selective drug compounds. However, the molecular generative models, specifically targeting scaffold hopping are relatively scarce. The primary objective of scaffold hopping is to identify compounds with distinct core structures while maintaining similar activities. This approach enables researchers to explore new lead compounds that may exhibit improved bioactivity and selectivity, while also bypassing existing intellectual property restrictions. Despite the potential benefits, the current methods for scaffold hopping remain limited and are in their early stages of development. The integration of scaffold hopping with artificial intelligence and generative models presents an opportunity to address these limitations. By leveraging advanced computational techniques and data-driven approaches, researchers can enhance scaffold-hopping capabilities and facilitate the discovery of novel drug candidates with desirable properties.

The DeepHop [12]method is regarded as a supervised translation task involving molecule-to-molecule transformations. Its objective is to construct pairs of molecules with similar 3D structures but distinct 2D structures using activity data sets from 40 kinases. The method employs a multimodal Transformer model that incorporates molecular sequence information, graph information, and protein information. However, this approach does not explicitly define the scaffold, making it challenging to generate molecules that preserve the side chains while solely modifying the scaffold. The SyntaLinker [13] and DeLinker [14] approaches are primarily fragment-based drug design methods that focus on generating a linker to connect two molecular fragments. While these methods touch upon the concept of scaffold hopping, they do not specifically target scaffold hopping as their main objective. Consequently, there is a lack of experimental validation specifically dedicated to scaffold hopping in these methods. The SyntaLinker Hybrid [15] method represents a combination of the SyntaLinker approach with the molecular fragment of the conserved kinase hinge region. By integrating these two components, the method aims to create kinase inhibitors with novel scaffolds by hybridizing the privileged fragment with the hinge region. This approach fundamentally relies on the principles and techniques of fragment-based drug design (FBDD). GraphGMVAE [16] is an innovative method for scaffold hopping in drug molecular design, developed by Tencent Laboratory. This approach leverages a graph-based Gaussian mixture hidden space variational autoencoder (GMVAE) to enable the generation of novel scaffolds with desirable properties. However, Tencent Laboratory hasn't been open-sourced, limiting its widespread application.

To enable scaffold hopping in molecule design, we propose an algorithm based on the framework of a variational autoencoder. Our algorithm aims to preserve the side chains while modifying the molecular scaffold. To achieve this, we adopt a strategy of separating the side-chain and scaffold embedding of the molecule. Specifically, we keep the side-chain embedding unchanged, while mapping the scaffold embedding to a mixture Gaussian distribution. This approach takes both scaffold and side-chain information into consideration during the scaffold generation process. Incorporating an automatic algorithm of adding side chains, our method performed scaffold hopping-guided molecular generation. To train our model, we perform pre-training on a large-scale ChEMBL dataset. We screen over 1 million molecules from the ChEMBL dataset and construct pre-training datasets using the ScaffoldGraph method for extracting molecular scaffolds. Additionally, we fine-tune the model using ScaffoldGraph extraction on datasets specific to molecules that exhibit activity against particular targets. This fine-tuning process aims to enhance the activity of the generated molecules for the target of interest. The effectiveness and superiority of our model are demonstrated through various evaluation metrics, including those commonly used in the field of drug design. Furthermore, we conduct case study analyses to provide insightful observations and validate the performance of our model.

## Materials and methods

### Data preparation

We retrieved over 1.9 million small molecules in canonical SMILES format from the ChEMBL database (version 31) [17]for our study. To ensure data quality, we performed preprocessing steps, including charge standardization, removal of small fragments and metals, and elimination of duplicates and invalid SMILES. The database was further refined by filtering based on molecular weight, heavy atom composition, medicinal chemistry filters, and PAINS filters. To extract the molecular scaffold from a molecule, the ScaffoldGraph [18] method was employed, though the Bemis-Murcko scaffold (BM scaffold) is more commonly utilized. ScaffoldGraph goes beyond the simple removal of substituents and performs a second-level extraction to capture core structural components more comprehensively. It not only enhances scaffold separation from the side chains but also enables a more thorough exploration of diverse scaffolds. The extracted scaffolds were then subjected to filtering based on well-defined criteria: (1) a minimum requirement of at least one ring (excluding benzene rings). The decision to remove the benzene ring is due to its ubiquitous occurrence in many molecules. If it were included, almost every molecule would contain a benzene ring, (2) a maximum limit of 20 heavy atoms, and (3) a constraint of no more than three rotatable bonds. Following the scaffold extraction and filtering processes on the extensive ChEMBL dataset, it is important to consider that a single molecule may correspond to multiple scaffolds. To address this, we randomly selected a representative scaffold for each molecule, resulting in the formation of a dataset comprising over 800,000 data pairs consisting of molecules and the corresponding scaffolds.

In this study, we carefully selected five distinct kinase proteins as cases for fine-tuning the pre-trained model, namely cyclin-dependent kinase 2 (CDK2), human epidermal growth factor receptor (EGFR), Janus kinase 1 (JAK1), Leucine-rich repeat kinase 2 (LRRK2), and Pim-1 proto-oncogene, serine/threonine kinase (PIM1). To obtain compounds with known bioactivity against these proteins, we extracted compounds with bioactivity (IC_50_, Ki) smaller than 10 micromoles from ChEMBL. Subsequently, scaffold extraction and scaffold filtering operations were performed on these molecules to isolate the underlying scaffolds. Given the limited amount of data available in the activity dataset, we retained all scaffolds that met the defined conditions, allowing for the possibility of one molecule corresponding to multiple scaffolds. The resulting datasets for the five kinases are summarized in Table 1.

**Table 1:** The data set the information of the five kinase proteins

Target protein	PDB ID	Uniprot ID	Number of data pairs
CDK2	1H00	P24941	1200
EGFR	2RGP	P00533	10,533
JAK1	6PTE	P23458	4860
LRRK2	7BJD	Q5S007	2436
PIM1	3UMW	P11309	3682

### Model architecture

**Fig. 1:**
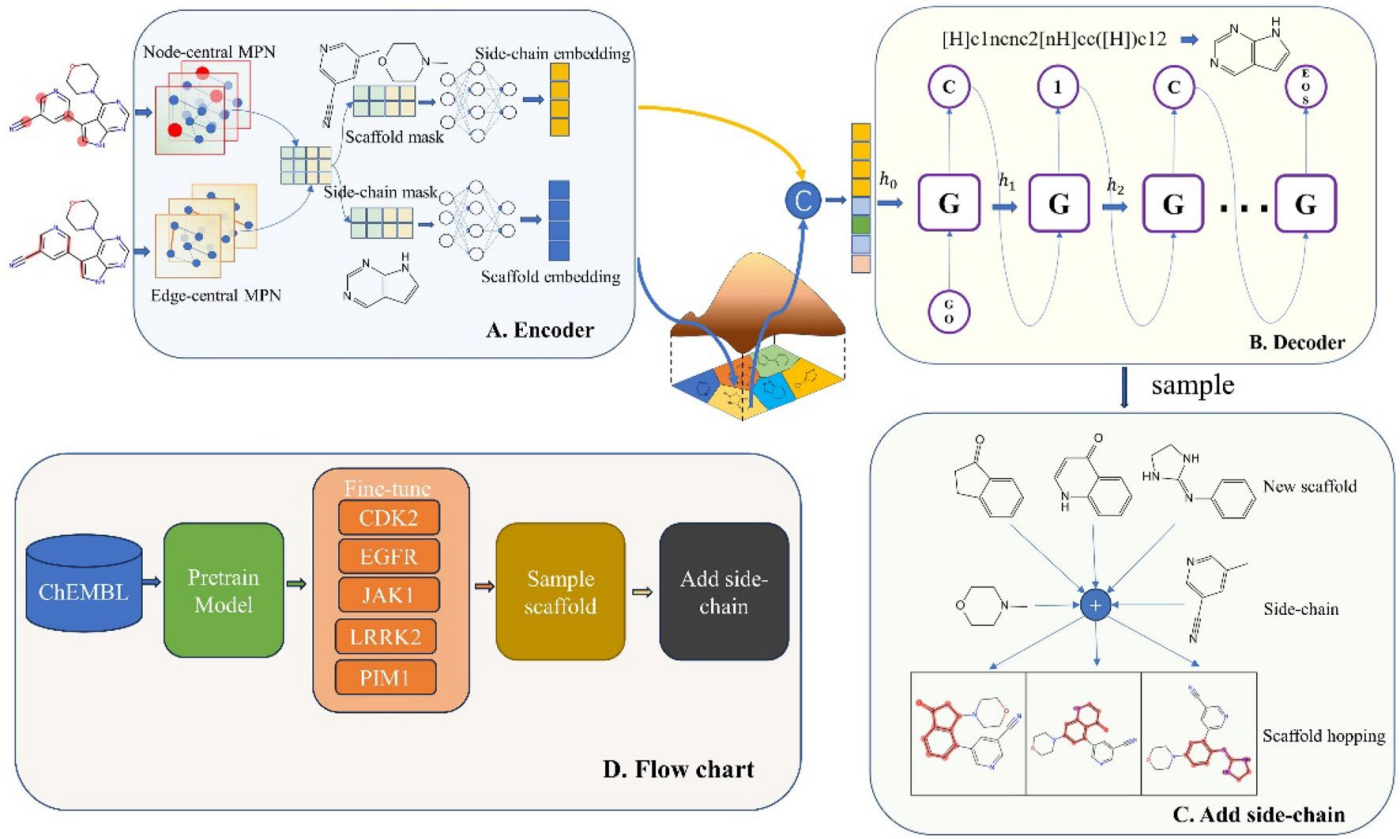
Model architecture diagram and workflow. **A** Multi-view graph neural network-based encoder. **B** RNN-based decoder for scaffold reconstruction and sampling. **C** Scaffold hopping: new scaffold sampling combined with reference molecule's side chains. **D** Flowchart depicting the training, sampling, and scaffold hopping of the model

The model architecture in our study is based on the concept of a variational autoencoder. However, unlike traditional VAE-based molecule generation methods, our focus lies specifically on scaffold generation to facilitate scaffold hopping in molecule design. To achieve this, we propose a novel variational autoencoder specifically designed for scaffold generation. The encoder (Fig. 1A) utilizes a multi-view graph neural network [19] to encode the edges (bonds) and nodes (atoms) of molecules separately, that is, perform message passing with nodes and edges as the center, respectively. In the readout phase, we concatenate the embeddings of nodes and edges together to obtain the whole molecular embedding. According to the scaffold of the molecule, the molecular embedding can be further divided into two parts, i.e., side-chain embedding and scaffold embedding. The scaffold embedding is projected onto a multivariate Gaussian mixture distribution, while the side-chain embedding remains unchanged. The decoder (Fig. 1B) employs a recurrent neural network (RNN) [20] model to concatenate the scaffold embedding with the side-chain embedding as the initial implicit vector, enabling the reconstruction of the scaffold SMILES. This scaffold generation process considers scaffold information and side-chain information of the original molecule.

### Encoder

The encoder in our model employs a graph messaging neural network [21] to effectively encode the molecular graph, as depicted in Fig. 1A. Each node in the graph is associated with a node eigenvector, which captures essential atomic properties such as atomic type, valency, and other relevant characteristics. Similarly, each edge in the graph is represented by a feature vector that encapsulates bond types.

The information transfer process based on molecular graph nodes is illustrated by Eq. (1). Here, ${h}_{v}^{0}$ represents the initial eigenvector of the node $v$, and ${h}_{v}^{l+1}$ represents the node feature vector after one iteration. The transfer of information from node $u$ to node $v$ is denoted by ${e}_{vu}$, where $u\in {N}_{v}$ represents that $u$ is a neighbor node of $v$. The activation function $\sigma (\cdot )$ is applied, and in our case, we utilize the rectified linear unit (ReLU), defined as $\mathrm{ReLU}(x) =\mathrm{ MAX}(0,x)$, as the activation function. The aggregation function used is $\mathrm{concat}(a,b)$. Equation (2) describes the information propagation among the edges of the molecular graph in the encoder network. The edge feature vector, ${h}_{vw}^{l+1}$, undergoes an iterative update from its initial value, ${h}_{vw}^{0}$. This update process takes into account the node features, ${x}_{u}$, associated with the connected nodes. The aggregation function combines relevant features, while the activation function introduces non-linearity to enhance the learning process. Equation (3) illustrates the process of propagating edge features to the nodes after L iterations. Equation (4) demonstrates the concatenation of the iterated node features with the edge features. This operation results in a matrix of size $n\times ({h}_{node} + {h}_{edge})$, where n represents the number of nodes, ${h}_{node}$ denotes the dimension of node eigenvectors, and ${h}_{edge}$ represents the dimension of edge eigenvectors. These mathematical formulations effectively capture the interplay between node and edge features, enabling a comprehensive representation of molecular structures.

1 $${h}_{v}^{l+1} =\, \sigma ({W}_{node}\left(\sum_{u\in {N}_{v}}concat\left({h}_{u}^{l} ,{e}_{vu}\right)\right)+{h}_{v}^{0}), {h}_{v}^{0}= \sigma ({W}_{nin}{x}_{v})$$

2 $${h}_{vw}^{l+1}=\,\sigma \left({W}_{edge}\left(\sum_{u\in {N}_{v }\backslash w}concat\left({h}_{uv}^{l},{x}_{u}\right)\right)+{h}_{vw}^{0}\right), {h}_{vw}^{0}=\sigma \left({W}_{ein}{e}_{vw}\right)$$

3 $${h}_{v}^{out}= \sigma ({W}_{eout}(\sum_{u\in {N}_{v}}concat({h}_{uv}^{L},{x}_{u})))$$

4 $${H}_{node}=concat({H}_{nout} , {H}_{eout})$$

To facilitate scaffold hopping, it is crucial to distinguish between the side-chain and scaffold parts within the molecule. This entails assigning a value of 1 to nodes belonging to the scaffold and 0 to nodes in the side chain. Consequently, we obtain a binary list, as depicted in Eq. (5), allowing us to separate the node features into ${H}_{side}$ and ${H}_{sca}$. This segregation enables a targeted focus on the scaffold characteristics, facilitating the generation of novel molecules with scaffold hopping techniques. To obtain fixed-length graph representations for ${H}_{side}$ and ${H}_{sca}$, a readout operation is performed. The readout function utilizes graph self-attention [22, 23], as illustrated in Eqs. (6) and (7). Equations (8) and (9) demonstrate the readout process for scaffold and side-chain embedding respectively. In these equations, the learnable matrices ${W}_{1} \in {R}^{{d}_{attn}\times {d}_{out}}$ and ${W}_{2}\in {R}^{{d}_{attn}\times r}$, with dimensions ${d}_{attn}\times {d}_{out}$ and ${d}_{attn}\times r$, respectively, play a crucial role. ${W}_{1}$ linearly transforms the node embedding from a dimensionality reduction space to a ${d}_{attn}$-dimensional space, while ${W}_{2}$ provides insights into the importance of $r$ nodes. The importance values are standardized using a softmax function. It is worth noting that ${W}_{1}$ and ${W}_{2}$ parameters are shared across scaffold and side-chain embedding. By applying the $\mathrm{Flatten}(\cdot )$ function, the graph-level embedding for the scaffold and side chain was obtained yielding fixed-length representation suitable for subsequent scaffold hopping tasks.

5 $${S}_{sca}=[1,\quad if \,i \in scaffold;\quad 0,\quad if\, i \in side]$$

6 $${Attention}_{sca}= softmax({W}_{1}tanh({W}_{2}{H}_{node}[{S}_{sca}]))$$

7 $${Z}_{sca} = Flatten( {Attention}_{sca}^{T} \cdot {H}_{node}[{S}_{sca}])$$

8 $${Attention}_{side}= softmax({W}_{1}tanh({W}_{2}{H}_{node}[{S}_{sca}^{-}]))$$

9 $${Z}_{side} = Flatten( {Attention}_{side}^{T} \cdot {H}_{node}[{S}_{sca}^{-}])$$

### Decoder

The decoder component of our model, depicted in Fig. 1B, utilizes an RNN-based language model. This design choice is inspired by the similarity between scaffold representation in SMILES format and sequential language in natural language processing. In language models, the ability to extract syntactic and semantic information enables the generation of coherent and meaningful sequences. Similarly, the RNN model in our decoder is capable of reconstructing the scaffold into canonical SMILES, considering the side-chain information. Additionally, the autoregressive nature of the RNN allows for scaffold sampling, facilitating the exploration of novel scaffold variations during the scaffold hopping process. we employ Gated Recurrent Unit (GRU) [20] units. These units play a crucial role in handling the sequential nature of SMILES strings. In our approach, we concatenate the side-chain embedding with the scaffold embedding to form the initial hidden state (h) of the RNN. This allows the model to consider both scaffold and side-chain information from the beginning of the generation process. During the training phase, we extract normalized scaffold SMILES and encode them as one-hot vectors, which serve as the target for reconstructing the scaffold SMILES. To facilitate the conversion of individual tokens into meaningful vector representations, we employ an embedding layer with 128 units. This layer converts each token into a 128-dimensional vector. The GRU component of our model comprises three layers, with each layer containing 512 neurons. These layers effectively capture the dependencies and patterns within the SMILES sequence. Finally, the output from the GRU layers is fed into a dense connection layer with the same number of neurons as the total number of words, including additional tokens indicating the start and end of the SMILES string.

10 $${f}_{t} = {\sigma }_{g}({W}_{f}{x}_{t} + {U}_{f}{h}_{t-1} + {b}_{f})$$

11 $${r}_{t} = {\sigma }_{g}({W}_{r}{x}_{t} +{U}_{r}{h}_{t-1} + {b}_{r})$$

12 $${\widehat{h}}_{t } = {\varnothing }_{h}({W}_{h}{x}_{t} + {U}_{h}({r}_{t} \odot {h}_{t-1}) + {b}_{h})$$

13 $${h}_{t} = (1 - {f}_{t}) \odot {h}_{t-1} + {f}_{t}\odot {\widehat{h}}_{t}$$

### Pre-training

The model undergoes a pre-training phase using a dataset consisting of over 800,000 pairs of molecular scaffolds extracted from the ChEMBL dataset. During pre-training, the model learns both the syntactic and semantic information encoded in molecular SMILES. Additionally, it enhances its understanding of scaffold-specific information and expands the range of scaffold types within its hidden representation. Before pre-training, a vocabulary is constructed by extracting words from the dataset, resulting in a total of 111 unique words. The standardized molecular scaffold SMILES are then one-hot encoded using this vocabulary, forming the target variable $X$. To reconstruct the scaffold, the information H encoded by the RNN is combined with the target variable to construct the reconstruction loss. The reconstruction loss is calculated using the cross-entropy loss function, as depicted in Eq. (14). Furthermore, a Kullback–Leibler (KL) [24] divergence loss function is employed to align the scaffold encoding with a multivariate normal distribution. This ensures that the scaffold encoding remains close to the normal distribution, as illustrated in Eq. (15). The two loss functions are combined using a weighted sum, as shown in Eq. (16), where β represents the weight assigned to balance the two losses. The weight β is adjusted incrementally with each Epoch to achieve a better balance between the reconstruction and KL divergence losses. Both the encoder and decoder are involved in the model training process, enabling the model to learn and capture the important features of molecular scaffolds.

14 $${L}_{recon} = cross\_entropy(X, H)$$

15 $${L}_{kl} = {D}_{kl}({q}_{\varphi }(z|X) || p(z))$$

16 $$Loss = {L}_{recon} + \beta {L}_{kl}$$

### Fine-tuning

The model undergoes a fine-tuning process using the known bioactive compounds against specific protein targets, namely CDK2, EGFR, JAK1, LRRK2, and PIM1. Since the number of active compounds is relatively small compared to the pre-trained dataset, all scaffolds that meet the specified conditions are retained. This means that one molecule may correspond to multiple scaffolds, which effectively expands the focused fine-tuning dataset. During fine-tuning, the learning rate is reduced compared to the pre-training phase. This adjustment enhances the model's ability to explore the chemical space of active compounds and improve its performance in generating novel molecules while maintaining or increasing activity. The loss function used in the fine-tuning procedure remains consistent with that employed during the pre-training phase. This ensures continuity in the optimization process and allows the model to further refine its capabilities in generating desired molecular structures.

### Sampling

The model performs scaffold sampling based on the molecular syntax and semantics learned from the training set, as well as the spatial distribution of molecules and scaffolds. Given a reference molecule and its corresponding scaffold, the model generates novel scaffolds that can replace the original one. The molecular scaffold and side chain are encoded using a graph-based neural network for information transfer. While the side chain remains unchanged, the scaffold embedding is resampled from the hidden space. In Eq. (17), the two embeddings are input into an RNN as initial hidden vectors. The scaffold is then sampled using the autoregressive property of the RNN model. Subsequently, the side chain of the reference molecule is assembled onto the newly sampled scaffold. This process results in a molecule with a novel scaffold. The splicing of the side chain is illustrated in Fig. 1C. The overall process of scaffold sampling and splicing, leading to scaffold hopping, is depicted in Fig. 1D. By leveraging the molecular syntax and spatial information, the model generates diverse scaffold replacements, enabling the exploration of novel chemical space for drug design and discovery.

17 $${\mathrm{h}}_{0} = concat({Z}_{side} ,\mu + {\sigma }^{2} \times N(\mathrm{0,1}))$$

### Principle of adding side chains

After the decoder outputs a scaffold, side chains need to be added back to the scaffold to obtain a complete generated molecule, as shown in Fig. 1C. Here, we use RDKit (https://rdkit.org/) tool to combine the scaffold with side chains and follow these simple principles: (1) Compare the sampled and original scaffold and enumerate all possible of side chain installation on the sampled scaffold; (2) Calculate the similarity of the topological fingerprint between the molecule after adding side chain and the original molecule; (3) Using the way to add side chain that ensures the generated molecule as much as possible similar to the original molecule; (4) Check the validity of the generated molecule, and if there are cases such as incorrect valence bonds or inability to connect side chain, the molecule is deemed invalid. Following these principles, the model ultimately could generate novel molecules with a hopped scaffold and invariant side chain.

### Baseline models

We compared our approach with the following baselines, The model was trained using the hyperparameters from the original paper, retrained on the ChEMBL dataset, and fine-tuned on corresponding activity datasets for five targets.

1. VAE [25] (Variational Autoencoder): The VAE is utilized for SMILES generation of molecules. It involves training two neural networks, namely the encoder and decoder. The encoder is responsible for reconstructing the SMILES representation of molecules, while the decoder maps the high-dimensional data representation of molecules to a latent space that follows a normal distribution. New molecules are generated by sampling from this latent space.
2. AAE [26](Adversarial Autoencoder): AAE addresses one of the main drawbacks of VAE, which is the limited applicability of the KL divergence term due to its closed-form analytical solution being available only for a few distributions. AAE combines the concepts of VAE and adversarial training, as seen in Generative Adversarial Networks.
3. LatentGAN [27]: LatentGAN combines autoencoders and adversarial neural networks. It involves pre-training a heterogeneous encoder on a ChEMBL dataset to capture molecular characteristics. Then, an adversarial network is trained to generate latent vectors that follow a desired distribution. Finally, the generated latent vectors are decoded using the heterogeneous encoder to obtain molecules.
4. QBMG [28]: QBMG is a drug molecule generation method based on a GRU recurrent neural network. It leverages the autoregressive property of RNNs to generate novel drug molecules. Additionally, it undergoes fine-tuning activity data sets specific to protein targets, enabling the generation of molecules that exhibit activity against those targets. This allows QBMG to generate novel drug candidates with desired properties for specific protein targets.
5. SyntaLinker [13]: SyntaLinker is a fragment-based drug design method that incorporates deep bar transformer neural networks. This approach utilizes the power of transformers to automatically establish connections between molecular fragments based on the knowledge learned from pharmaceutical chemistry databases. Moreover, the model is capable of performing scaffold hopping, enabling the generation of structurally diverse compounds.
6. REINVENT2[35]: REINVENT2 is an advanced RNN-based molecular design model known for its ability to generate diverse and innovative chemical compounds. In this study, we employed REINVENT2 with a scaffold penalty in the reinforcement learning process to generate new molecules referring to the given reference compounds.

### Evaluation metrics

The performance of these generative models is evaluated using two sets of evaluation metrics. The first set of metrics is the same as MOSES [29], which is commonly used in the field of molecular generation to evaluate the ability to generate valid and chemically diverse druglike molecules. We named these metrics as general generative model evaluation metrics (GEM) which include:

1. Validity: the proportion of generated molecular SMILES that can be parsed and validated by RDKIT.
2. Uniqueness1K: the proportion of unique and valid molecules within the top 1 K generated molecules.
3. Uniqueness5K: the proportion of unique and valid molecules within the top 5 K generated molecules.
4. Filter: the proportion of molecules generated by the model that can pass through MOSES using Filter when constructing data sets: the molecules were filtered via custom medicinal chemistry filters (MCFs) and PAINS filters etc.
5. Novelty: the proportion of generated molecules that are not in the training sets.
6. Scaffold uniqueness: the proportion of unique and valid scaffold within the generated molecules.
7. Scaffold novelty: the proportion of scaffolds of generated molecules that are not in the training sets.

In addition, another more important set of metrics is utilized to assess the ability of these models to generate new molecules that not only satisfy the scaffold hopping requirement but also retain the desired activity. In the context of designing drugs through scaffold hopping, medicinal chemists aim to obtain a novel compound with a new scaffold while maintaining similar activity, even if it is slightly lower. Therefore, it is crucial for these models to effectively generate molecules that fulfill both criteria to be considered successful tools for drug design. In this study, we have made use of both GraphDTA [30] and LeDock [31](http://www.lephar.com) to predict activity scores. The reason for this is that GraphDTA utilizes a deep learning method to predict the activity score, while LeDock uses a conventional molecular docking approach. By referring to both these two methods, we can obtain a more comprehensive understanding of the activity scores of the molecules generated by the model. Specifically, the following four metrics are used in this work, and we named them scaffold hopping generative model evaluation metrics (SEM):

(8) Active mean: the average activity score of the molecules generated by the model.
(9) Active rate: the proportion of generated molecules that have activity scores better than the corresponding reference compound.
(10) Hop rate: the proportion of generated molecules that satisfy the criteria for scaffold hopping, which involves retaining the side chain while introducing a hopped scaffold.
(11) Success rate: the proportion of generated molecules that satisfy both the requirements for scaffold hopping and retaining or increasing activity score compared to the reference molecule.

## Results and discussion

The complete workflow of our model in this study is shown in Fig. 1D. Initially, we collected data from the ChEMBL database and followed a rigorous data preparation procedure to obtain 800,000 molecule-scaffold pairs for model pre-training. Additionally, we gathered known active compounds against five kinase proteins, namely CDK2, EGFR, JAK1, LRRK2, and PIM1, as shown in Table 1. Next, we fine-tuned the pre-trained model on the active compound dataset for each of the five targets. To ensure the robustness of the model and obtain a statistical comparison of its performance, we selected 20 different compounds as reference compounds from the known active compound dataset for each kinase. We pre-defined the scaffold of the reference compound that required hopping based on the pharmacophore core structure of the compound near the hinge binder. This was done because the structure binding to the hinge region is the most significant part of the design of kinase inhibitors. All the identified scaffolds were provided in the Additional file 1: Fig. S1–S5. Using the fine-tuned model, we sampled 5000 novel scaffolds and installed them to molecules according to the principle of adding a side chain for each reference compound. This resulted in 100,000 generated molecules obtained for each kinase. Finally, we evaluated the generated molecules using established eleven metrics, including seven GEM and four SEM.

To ensure a fair comparison, we trained baseline models and utilized them for molecule generation. The VAE, AAE, LatentGAN, and QBMG models were pre-trained and fine-tuned on five targets, following the MOSES [29] framework's proposed pipeline. We retrained these models using their respective papers' specified hyperparameters. To maintain consistency with our model's generation tasks, we independently sampled these models 20 times for each target to ensure an equal comparison among these baselines. Consequently, we generated 100,000 molecules against each target for each model. The SyntaLinker required inputting two fragments to generate the linker. As we aimed to produce new scaffolds, we divided the reference compound into two fragments according to the pre-defined scaffold and removed it. Following this step, SyntaLinker generated 5000 molecules for each reference compound.

### Model performance on GEM

We first assessed the performance of our model on seven GEM, namely Validity, Uniqueness1k, Uniqueness5k, Novelty, Filtering, Scaffold Uniqueness, and Scaffold Novelty. We evaluated the model against five targets, with a total of 100,000 molecules sampled and tested for each target. The results were obtained by averaging the performance across 20 reference compounds for each target. As shown in Table 2, our model demonstrated acceptable validity and uniqueness metrics of 90% and 60%, respectively, indicating that it has learned the representation of chemical molecules well and can be used for de novo design. Furthermore, our model's robustness across different targets highlights its reliability. Our multi-view graph network variational autoencoder model for a molecular generation was also shown to achieve a validity and uniqueness of 99% in an ablation experiment. Additionally, our model's novelty of scaffold and molecule was better than most baseline models, with the novelty of molecule reaching 100% for all targets. These results demonstrate that our model is capable of exploring the unseen chemical space and generating novel molecules that are distinct from known compounds. Overall, our model's performance on various metrics highlights its ability to generate valid and novel molecules through scaffold hopping.

**Table 2:** The performance of our model on general generative model evaluation metrics (GEM) among five distinct targets: CDK2, EGFR, JAK1, LRRK2, and PIM1

Protein	Validity $\uparrow$	Uniqueness1K $\uparrow$	Uniqueness5K $\uparrow$	Filter $\uparrow$	Scaffold uniqueness $\uparrow$	Scaffold novelty $\uparrow$	Novelty $\uparrow$
CDK2	0.9047	0.5989	0.4423	0.8186	0.4880	0.4853	1.0000
EGFR	0.9017	0.6279	0.4762	0.5976	0.5220	0.4989	1.0000
JAK1	0.8967	0.5987	0.4434	0.9059	0.4937	0.4921	1.0000
LRRK2	0.9022	0.6021	0.4457	0.8957	0.4932	0.4748	1.0000
PIM1	0.8949	0.5982	0.454	0.8208	0.5028	0.4898	1.0000

To better understand the estimation of how similar the proposed novel scaffolds are to the molecules the known compounds for the targets (CDK2, JAK1, EGFR, LRRK2, and PIM1) in the ChEMBL database. we evaluated the chemical space coverage by calculating the ECFP fingerprint used as a t-distributed Stochastic Neighbor Embedding (t-SNE) visualization. As Additional file 1: Fig. S6 shows in the t-SNE plot, the generated molecules are not only capable of scaffold hopping around the reference molecules but also exploring the chemical space of scaffolds to select appropriate scaffolds for hopping.

### Model performance on SEM

The main task of this study is molecular generation through scaffold hopping, thus, the model performance on SEM is especially significant. Here, we conduct a more comprehensive performance comparison between our model and baseline models, involving the activity scores of generated molecules. As introduced in the section of Materials and Methods, SEM includes four metrics, namely active mean, active rate, hop rate, and success rate. The activities of molecules are evaluated using two prediction methods: GraphDTA [29] and LeDock [31] (http://www.lephar.com). GraphDTA predicts drug-target activity based on the protein sequence using a deep learning method, while LeDock utilizes the protein structure and conducts molecular docking to predict the binding affinity score. As a validation, we utilized GraphDTA to predict the activity scores (IC_50_ or Ki) of the reference compounds against five targets, and the RMSE of prediction is 0.88 (Additional file 1: Fig. S7) which is well acceptable. Thus, by referring to both these two methods, we can obtain a more comprehensive understanding of the activity scores of the molecules generated by the model. Only the molecule that satisfies both the requirements for scaffold hopping and retaining or increasing activity score compared to the reference molecule is considered a success molecule, so we underline that the success rate is the most important metric to evaluate model performance for scaffold hopping of molecule.

The performance comparison between our model and baseline models on SEM among five distinct targets is shown in Table 3 (For aesthetic purposes, Table 3 does not include the portion with standard deviations. A table with the standard deviation part, like Additional file 1: Table S6, is available.). It is highlighted that our model reaches the highest success rate for all five targets either evaluated by GraphDTA or evaluated by LeDock. Specifically, the hop rate of our model is near 100% for all targets, while the other methods are all smaller than 40%. Although REINVENT2 with a scaffold penalty in the reinforcement learning can generate a greater variety of novel molecular scaffolds, its success rate remains low due to the difficulty in satisfying side-chain constraints with the generated molecules. Even the linker design method, SyntaLinker, only has a hop rate of approximately 30%. We speculate that it is the multi-view graph neural network and Gaussian mixture sampling in our model that facilitates good performance for scaffold hopping. And for the activity evaluations, the ligand-based methods such as VAE, AAE, and QBMG perform better not surprisingly, because their feature of generating molecules is to imitate known active molecules and can only generate molecules within similar chemical space (the fact of lack of novelty can prove this point). Compared to these methods, the performance of our model and SyntaLinker are slightly inferior, because of the additional molecular side chains constraints when generating molecules. Nevertheless, our model presents the active rate better than 60%, and for LRRK2 and PIM1 the active rate even reaches 92.4% and 94.3, respectively. The impressive active rate is probably contributed by the joint embedding of the scaffold and side chain. In our model, we have leveraged the concatenated embedding of scaffold and side chain to enable our variational autoencoder to efficiently sample potential scaffold while simultaneously considering the side chain. Without the side-chain embedding, the model performs not that well, as shown in the subsection of the ablation experiment (Model 3).

**Table 3:** The performance comparison between our model and baseline models on scaffold hopping generative model evaluation metrics (SEM) among five distinct targets: CDK2, JAK1, EGFR, LRRK2, and PIM1

Protein	Model	SAscore	GraphDTA				Ledock			
			Active mean	Active rate	Hop rate	Success rate	Active mean	Active rate	Hop rate	Success rate
CDK2	AAE	**2.796**	7.559	0.787	0.005	0.007	**−** **9.522**	**0.950**	0.016	0.012
	VAE	2.934	**8.006**	**0.992**	0.003	0.003	− 9.335	0.928	0.014	0.009
	LatentGAN	3.226	7.610	0.863	0.007	0.005	− 9.182	0.882	0.009	0.009
	QBMG	2.860	7.873	0.971	0.003	0.003	− 9.339	0.911	0.010	0.008
	SyntaLinker	2.933	6.753	0.469	0.296	0.113	− 8.164	0.559	0.314	0.176
	REINVENT2	3.230	7.205	0.595	0.128	0.085	− 8.966	0.866	0.027	0.027
	our	3.042	7.151	0.676	**1.000**	**0.676**	− 8.208	0.622	**1.000**	**0.622**
EGFR	AAE	**2.672**	8.295	0.929	0.007	0.006	− 10.880	0.947	0.003	0.003
	VAE	2.738	**8.304**	**0.948**	0.008	0.008	− 10.960	0.945	0.002	0.002
	LatentGAN	2.866	7.901	0.872	0.003	0.003	− 10.320	0.913	0.002	0.002
	QBMG	2.720	8.221	0.941	0.006	0.006	**−** **10.980**	**0.952**	0.002	0.001
	SyntaLinker	2.764	6.895	0.506	0.337	0.167	− 8.480	0.721	0.366	0.331
	REINVENT2	2.890	7.187	0.703	0.208	0.156	− 9.505	0.878	0.213	0.212
	our	2.949	7.018	0.613	**1.000**	**0.613**	− 8.874	0.923	**1.000**	**0.923**
JAK1	AAE	3.106	8.014	0.627	0.001	0.001	**−** **8.979**	0.996	0.001	0.001
	VAE	3.502	**8.972**	**1.000**	0.063	0.063	− 8.797	0.996	0.010	0.010
	LatentGAN	4.003	7.984	0.613	0.001	0.001	− 8.778	0.970	0.001	0.001
	SyntaLinker	3.460	7.196	0.283	0.215	0.053	− 7.331	0.498	0.233	0.109
	QBMG	**3.439**	8.920	**1.000**	0.053	0.053	− 8.957	**1.000**	0.012	0.012
	REINVENT2	3.307	6.862	0.300	0.077	0.062	− 8.504	0.871	0.001	0.001
	our	3.510	7.739	0.510	**0.952**	**0.462**	− 7.861	0.721	**0.955**	**0.676**
LRRK2	AAE	**2.682**	7.109	**1.000**	0.062	0.062	− 8.471	0.992	0.064	0.064
	VAE	2.722	7.177	**1.000**	0.033	0.033	− 7.888	0.991	0.065	0.065
	LatentGAN	2.880	6.981	0.988	0.042	0.042	− 7.878	0.986	0.072	0.072
	SyntaLinker	2.872	6.542	0.860	0.355	0.295	− 6.980	0.839	0.373	0.336
	QBMG	3.396	**7.485**	**1.000**	0.072	0.072	**−** **8.562**	0.950	0.028	0.022
	REINVENT2	2.938	6.836	0.973	0.245	0.244	**−** **8.400**	**1.000**	0.242	0.242
	our	2.915	6.663	0.924	**1.000**	**0.924**	− 7.205	0.928	**1.000**	**0.928**
PIM1	AAE	**2.684**	8.179	0.893	0.007	0.007	**−** **7.869**	0.988	0.005	0.005
	VAE	2.803	**9.012**	**0.971**	0.013	0.011	− 7.582	0.986	0.016	0.015
	LatentGAN	2.949	8.059	0.885	0.016	0.015	− 7.454	0.956	0.014	0.014
	SyntaLinker	2.938	7.329	0.622	0.239	0.129	− 6.626	0.645	0.242	0.142
	QBMG	2.779	8.648	0.949	0.013	0.012	− 7.661	0.991	0.012	0.012
	REINVENT2	3.095	7.930	0.834	0.193	0.154	− 7.699	**0.992**	0.172	0.172
	our	3.172	8.310	0.943	**1.000**	**0.943**	− 6.879	0.796	**1.000**	**0.796**

The best 10% of molecules generated by each model were evaluated. For each metric, the best result among all baseline models is represented as bold format

**Fig. 2:**
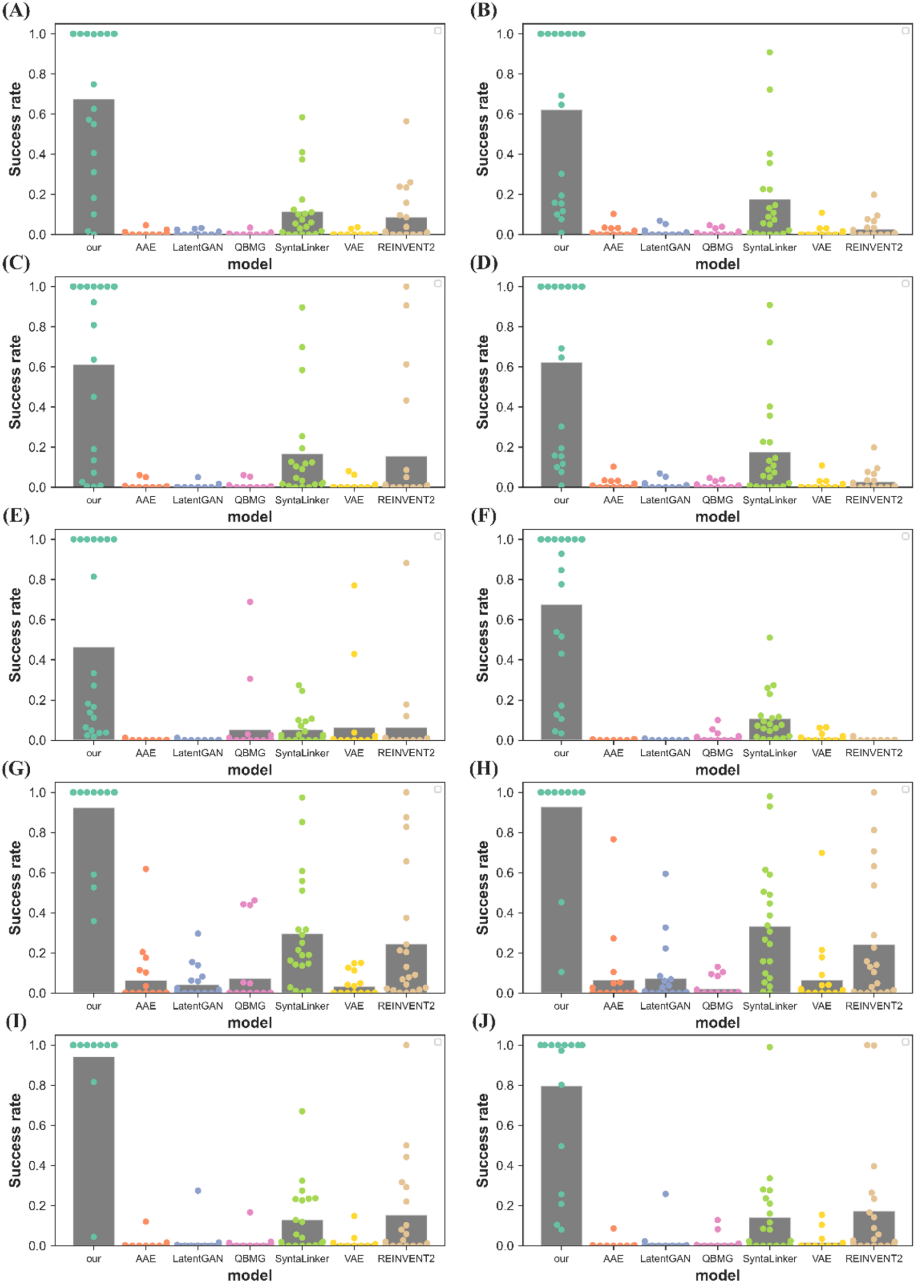
The success rate of the best 10% molecules evaluated by GraphDTA and LeDock represented as a swarm plot. Swarm plot of success rate evaluated by GraphDTA for 20 reference compounds among the target of **A** CDK2; **C** EGFR; **E** JAK1; **G** LRRK2; **I** PIM1. Swarm plot of success rate evaluated by LeDock for 20 reference compounds among the target of **B** CDK2; **D** EGFR; **F** JAK1; **H** LRRK2; **J** PIM1. The points denote the success rate of generated molecules, and the gray bars are the average success rate for 20 reference compounds

The results of our model are highly encouraging. Not only does it exhibit acceptable activity performance, but it also outperforms the baseline models in terms of scaffold hopping performance. As a result, the overall success rate of our model is significantly better than that of the baseline models. Figure 2 shows it more vividly, while other methods show a success rate near zero except SyntaLinker and REINVENT2, and our model has the best success rate among five distinct targets. Additionally, we also assess the synthetic difficulty of the generated molecules through SA score calculated by RDKit, the results demonstrate that all these molecular generative models are capable of generating easy-to-synthesize molecules (the average SA scores are all smaller than 4 as shown in Table3). These findings are a testament to the effectiveness of our approach and the potential it holds for future research in the field. However, there is still room for improvement in our current model. For example, although the overall success rate is high, there are still some reference compounds with low success rates. Further improvement may extend its capabilities to deal with those special reference compounds.

### Ablation experiment

In this subsection, we conducted ablation experiments on our model to investigate the impact of different components on success rates. Specifically, our model is based on a multi-view graph neural network, namely combines the node-central message passing network (node-central MPN) and edge-central message passing network (edge-central MPN), which enables information propagation from both the edge and node perspectives. The remove of node-central MPN (Model 1) and edge-central MPN (Model 2) will be adopted as the ablation experiments, respectively. Additionally, the variational autoencoder in our model is used to encode the molecule and decode the scaffold, which is different from the general molecule-to-molecule generative methods. There are two reasons that we adopted this strategy but did not choose scaffold-to-scaffold or molecule-to-molecule encode-decode strategies. Firstly, scaffold to scaffold encode-decode strategy will lose the information of side chains, and it is hard to ensure generating scaffolds that are suitable for the side chains. Secondly, molecule to molecule encode-decode strategy could not promise scaffold hopping and retaining the side chain simultaneously. As for comparison, these two strategies were also tested in our ablation experiments, corresponding without side-chain embedding (Model 3) and without side-chain adding (Model 4), respectively. Finally, the Gaussian mixture distribution involved in our model is significantly important for scaffold hopping. Model 5 is the ablate model without the Gaussian mixture distribution, namely a general graph-based VAE. And Model 6 is our model that involves all these components.

The ablation experiments conducted in this study are summarized in Table 4. The results reveal that the performance of the model with a missing node-central MPN (Model 1), edge-central MPN (Model 2), or side-chain embedding (Model 3) is slightly inferior to that of the complete model (Model 6). Furthermore, the direct molecule-to-molecule generative model without the side-chain-adding strategy (Model 4) exhibits poorer performance compared to our molecule-to-scaffold model that incorporates a side-chain-adding step (Model 6). Notably, Model 5 performs worse than all other ablate models, with a success rate of only about 10%, which is comparable to the baseline models of VAE, AAE, etc. This is because Model 5 is essentially a graph VAE model. These findings provide valuable insights into the importance of each component in our proposed model and highlight the significance of incorporating a side-chain adding step in the molecule to scaffold the generation process.

**Table 4:** shows the results of the ablation experiments

Model	Node-central MPN	Edge-central MPN	Side-chain embedding	Side-chain adding	Gaussian mixture distribution	LeDock Success rate	GraphDTA Success rate
1		√	√	√	√	0.458 ± 0.206	0.536 ± 0.299
2	√		√	√	√	0.656 ± 0.240	0.604 ± 0.339
3	√	√		√	√	0.565 ± 0.378	0.721 ± 0.350
4	√	√	√		√	0.259 ± 0.231	0.250 ± 0.248
5	√	√	√			0.014 ± 0.043	0.019 ± 0.070
6	√	√	√	√	√	**0.720 ± 0.326**	**0.776 ± 0.333**

For each metric, the best result among all baseline models is represented as bold format

### De novo* molecular design *via* scaffold hopping*

**Fig. 3:**
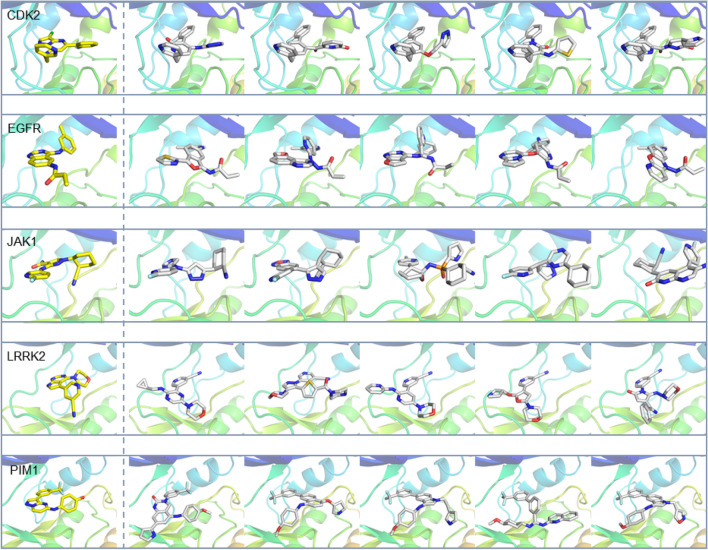
De novo molecular design via scaffold hopping for the target of CDK2, EGFR, JAK1, LRRK2, and PIM1. The last five columns are generated molecules, and the first column is the reference molecule

In this study, we introduced a novel method for de novo molecular design via scaffold hopping, termed ScaffoldGVAE. The previous model performance on GEM and SEM, and the comprehensive comparison between the baseline models have demonstrated the potential of our proposed model in de novo molecular generation tasks. In this section, for each target, we took one reference compound as an example to further analyze the qualities of the generated molecules from the perspective of 3D-structure docking poses. As represented in Fig. 3, five randomly selected generated molecules and the reference compound are displayed. The docking poses of the generated compounds are almost aligned with the reference compound, and the binding pocket is conserved. Furthermore, most of the generated new compounds exhibited better binding affinity as evaluated by the LeDock docking score and GraphDTA score. Notably, all the molecules generated in this study have different scaffolds from the reference compound. The ability to preserve or even enhance the activity of the molecule with a different scaffold demonstrates the superiority of our method.

### Design of LRRK2 inhibitors based on multiple reference compounds

Parkinson's disease (PD) [32] is a neurodegenerative disorder that affects millions of people worldwide, and current treatments only provide symptomatic relief. LRRK2 has been identified as a key player in the pathogenesis of PD, and inhibiting its activity has the potential to slow or even halt disease progression [33]. Thus, the development of potent and selective inhibitors is of great importance. To achieve this goal, we utilized ScaffoldGVAE, our new method for generating small molecules via scaffold hopping, aimed to design some potential inhibitors of LRRK2.

**Fig. 4:**
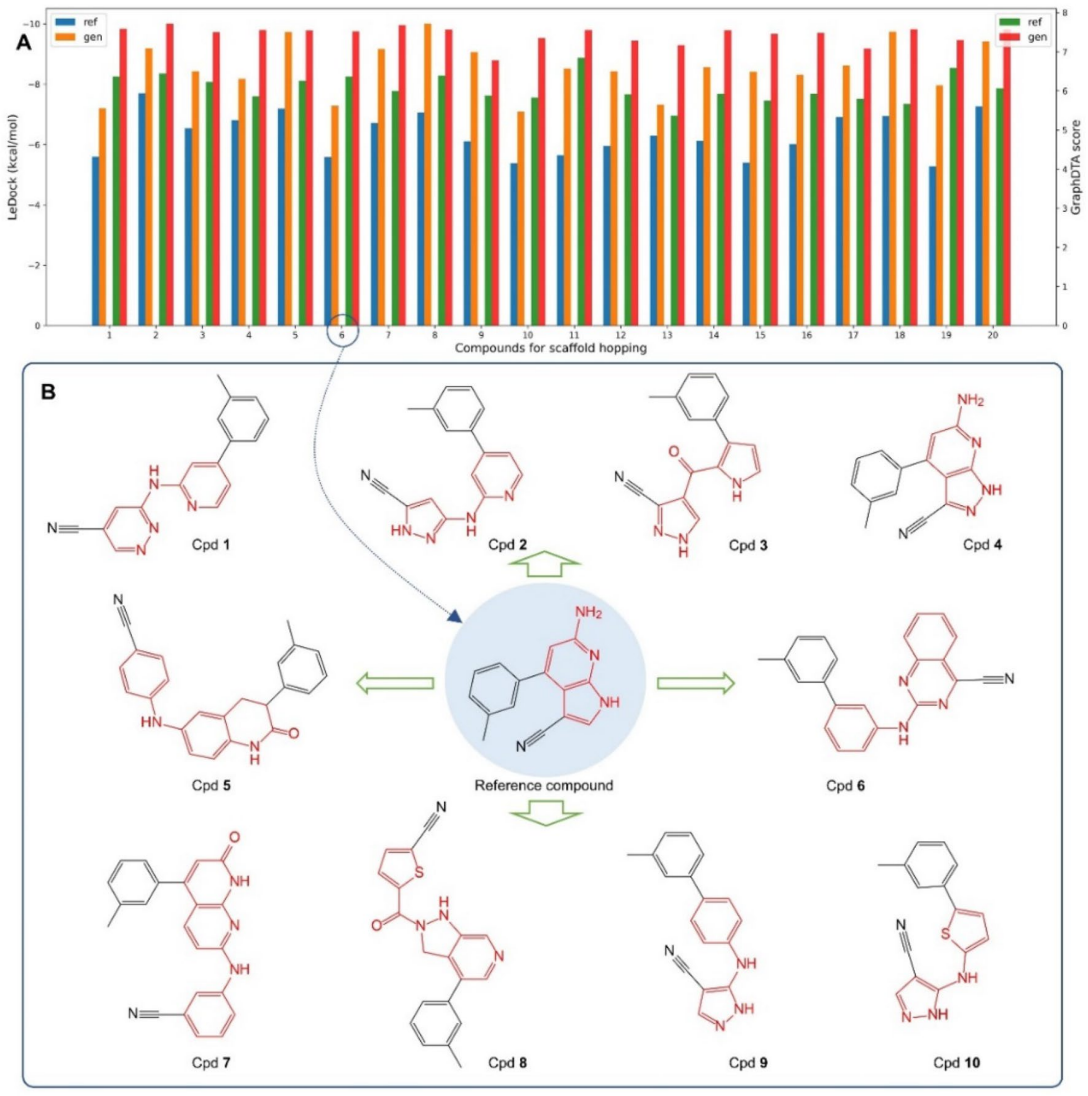
The performance of ScaffoldGVAE in generating LRRK2 inhibitors and the example generated compounds. **A** Comparison of the reference compound and the corresponding top1 generated molecule on the performance of LeDock docking score and GraphDTA score. The blue and orange bars represent the docking score of LeDock, while the green and red bars represent the GraphDTA score. A total of 20 different reference compounds for scaffold hopping were tested, and **B** listed ten example compounds generated via scaffold hopping using the 6^th^ compound as a reference

To evaluate the effectiveness of ScaffoldGVAE in generating LRRK2 inhibitors, we compared the docking and GraphDTA scores of reference compounds with those of the top1 generated molecule. The results, as shown in Fig. 4A, demonstrate that ScaffoldGVAE was able to generate compounds with higher scores than the reference compounds. This suggests that our method is capable of generating novel compounds with potential inhibitory activity against LRRK2.

To further validate our approach, we depicted ten example compounds using the 6th compound of LRRK2 as a reference, which is listed in Fig. 4B. These compounds were randomly selected for display and were not necessarily the top molecules by ranking. However, the statistical analysis of their docking and GraphDTA scores (see Table 3) showed that most of these generated compounds have similar or better scores compared to the reference compounds. This demonstrates the effectiveness of ScaffoldGVAE in generating novel compounds with potential inhibitory activity against LRRK2.

It is worth noting that the aminopyridine pyrrole structure is the core scaffold of the reference compound that binds to the hinge of the LRRK2 [34] kinase domain. The reference compound has bioactivities of 471 nM and 69.18 nM for inhibiting human LRRK2 A2016T and G2019S mutant phosphorylation at ser935 transfected in HEK293 cells. Additional file 1: Fig. S8 displays the binding poses of the reference compound, which show that it binds to the hinge with three hydrogen bonds. Furthermore, Fig. 4B demonstrates that the new scaffolds identified from scaffold hopping are mostly bioisosteres, consistent with the principles of medicinal chemistry. These results demonstrate the effectiveness of ScaffoldGVAE in generating novel compounds with potential inhibitory activity against LRRK2. The ability to generate new compounds with different scaffolds while retaining potential interaction with the hinge binder provides a promising approach for designing potential LRRK2 inhibitors.

### In silico* validation of the generated LRRK2 inhibitors through MM/GBSA*

**Fig. 5:**
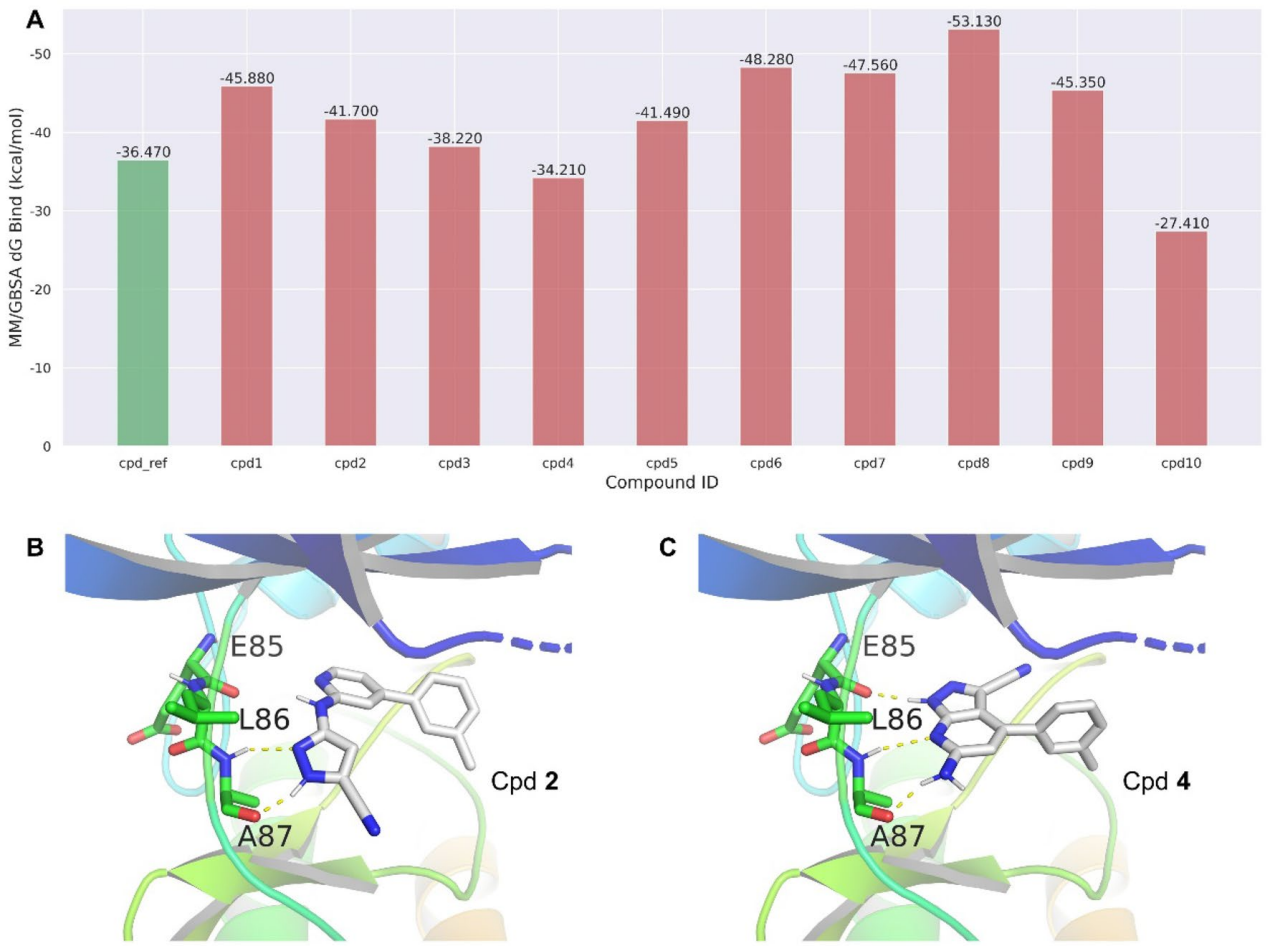
Binding free energy modeling of the generated LRRK2 inhibitors. (**A**) The MM/GBSA binding free energy of the reference compound (green bar) and the corresponding ten example-generated compounds (red bar). **B**, **C** are the binding modes of Cpd **2** and Cpd **4**, respectively. The residues of the hinger binder are shown as sticks, and the yellow dot line represents the hydrogen bond interaction

Molecular mechanics/generalized Born surface area (MM/GBSA) is a widely used method for predicting the binding free energy of protein–ligand complexes. This method involves the calculation of the potential energy of the protein–ligand complex using molecular mechanics force fields and the solvation energy using a continuum solvent model. This subsection aimed to use the in silico MM/GBSA method to validate the compounds generated by ScaffoldGVAE. We wanted to determine whether the generated compounds have favorable binding energies and whether they are likely to bind to the LRRK2 protein. The results are shown in Fig. 5. As can be seen from the figure, most of the generated compounds have favorable binding energies, ranging from − 40 to − 60 kcal/mol. This indicates that these compounds are likely to bind to the LRRK2 protein with high affinity. We also analyzed the binding modes of Cpd **2** and Cpd **4** with lower and higher binding energies compared to the reference compound, respectively. As shown in Fig. 5B, C, the results showed that these compounds interact with the key residues of the LRRK2 protein, such as Glu85, Leu86, and Ala87. These residues are known to be important for the binding of LRRK2 inhibitors and are often targeted by existing drugs. Notably, Cpd **4** share the same binding mode as the reference compound, while Cpd **2** induce a new binding mode that mainly interacts with Ala87 through two strong hydrogen bonds, and the side chains totally turnover compared to Cpd **4**. These illustrated that ScaffoldGVAE can not only generate compounds that preserve the original binding mode but also is capable of identifying new bind modes. Our results show that most of the generated compounds have favorable binding energies and are likely to bind to the LRRK2 protein with high affinity. These compounds can be further optimized and synthesized for in vitro testing, which may lead to the discovery of novel drugs for treating Parkinson's disease and other related disorders.

## Conclusion

In this study, we proposed the ScaffoldGVAE model, an advanced model specifically designed for drug molecule scaffold hopping. The model is based on the architecture of a variational autoencoder, where the encoder component utilizes a state-of-the-art multi-view graph neural network. This neural network considers both edge-central message passing and node-central message passing, thereby enhancing the information propagation capability of the encoder. The decoder employs an RNN model to decode the latent vectors into scaffold SMILES representations. Additionally, we introduced an algorithm for automatically adding the side chain.

The ScaffoldGVAE model, along with several baselines, was pre-trained on the ChEMBL dataset and then fine-tuned on five target activity datasets: CDK2, EGFR, LRRK2, JAK1, and PIM1. The model performances were evaluated on seven general generative model evaluation metrics (GEM) and four scaffold hopping generative model evaluation metrics (SEM). The results demonstrate that our proposed model is capable of exploring the unseen chemical space and generating novel molecules that are distinct from known compounds. Additionally, our model not only exhibits acceptable activity performance but also outperforms the baseline models in terms of scaffold hopping performance. Further ablation experiments provide valuable insights into the importance of each component in our proposed model and highlight the significance of incorporating a side-chain adding step in the molecule to scaffold the generation process.

These findings are a testament to the effectiveness of our approach. Further investigation of the performance from the perspective of 3D-structure docking poses, illustrated the model’s ability to generate molecules that preserve or even enhance the activity of the molecule with a different scaffold. Considering the good performance of our model mentioned above, we employed it in the design of LRRK2 inhibitors, and the designed molecules were in silico validated by MM/GBSA. These compounds can be further optimized and synthesized for in vitro testing, which may lead to the discovery of novel drugs for treating Parkinson's disease and other related disorders. As a result, it demonstrates the effectiveness of ScaffoldGVAE in generating novel compounds with potential inhibitory activity against LRRK2. This novel approach we developed can be applied to other proteins and diseases, thereby contributing to the future development of new drugs.

### Supplementary Information

**Additional file 1: Fig. S1**. Reference molecules and scaffolds on the CDK2 protein. **Fig. S2**. Reference molecules and scaffolds on the EGFR protein. **Fig. S3**. Reference molecules and scaffolds on the JAK1 protein. **Fig. S4**. Reference molecules and scaffolds on the LRRK2 protein. **Fig. S5**. Reference molecules and scaffolds on the PIM1 protein. **Table S1**. The result of the AAE model on general generative model evaluation metrics (GEM). **Table S2**. The result of the VAE model on general generative model evaluation metrics (GEM). **Table S3**. The result of the LatentGAN model on general generative model evaluation metrics (GEM). **Table S4**. The result of the QBMG model on general generative model evaluation metrics (GEM). **Table S5**. The result of the SyntaLinker model on general generative model evaluation metrics (GEM). **Fig. S6**. Chemical space of generated molecules and bioactive ligands of five distinct targets: (A) CDK2, (B) JAK1, (C) EGFR, (D) LRRK2, and (E) PIM1 visualized by t-SNE dimensionality reduction. **Fig. S7**. Correlation between the experimental activity values of 100 reference molecules and the activity values predicted by GraphDTA. **Table S6 and S7**. The performance comparison between our model and baseline models on scaffold hopping generative model evaluation metrics (SEM) among five distinct targets: CDK2, JAK1, EGFR, LRRK2, and PIM1. The best 10% and 30% molecules generated by each model were evaluated. **Fig. S8**. The success rate of the best 30% molecules evaluated by GraphDTA and LeDock represented as a swarm plot. Swarm plot of success rate evaluated by GraphDTA for 20 reference compounds among the five targets. **Fig. S9**. The binding poses of the reference compound against LRRK2. The yellow dot line denotes the hydrogen bond.)

## Data Availability

Demo, instructions, and codes for ScaffoldGVAE are available at https://github.com/ecust-hc/ScaffoldGVAE
