# Supplementary material for: ScaffoldGVAE: scaffold generation and hopping of drug molecules via a variational autoencoder based on multi-view graph neural networks
Source: J Cheminform. 2023 Oct 4;15:91. doi: 10.1186/s13321-023-00766-0 (PMC10548653; doi:10.1186/s13321-023-00766-0)
Supplement: Supplementary file 1 — Additional file 1: Fig. S1. Reference molecules and scaffolds on the CDK2 protein. Fig. S2. Reference molecules and scaffolds on the EGFR protein. Fig. S3. Reference molecules and scaffolds on the JAK1 protein. Fig. S4. Reference molecules and scaffolds on the LRRK2 protein. Fig. S5. Reference molecules and scaffolds on the PIM1 protein. Table S1. The result of the AAE model on general generative model evaluation metrics (GEM). Table S2. The result of the VAE model on general generative model evaluation metrics (GEM). Table S3. The result of the LatentGAN model on general generative model evaluation metrics (GEM). Table S4. The result of the QBMG model on general generative model evaluation metrics (GEM). Table S5. The result of the SyntaLinker model on general generative model evaluation metrics (GEM). Fig. S6. Chemical space of generated molecules and bioactive ligands of five distinct targets: (A) CDK2, (B) JAK1, (C) EGFR, (D) LRRK2, and (E) PIM1 visualized by t-SNE dimensionality reduction. Fig. S7. Correlation between the experimental activity values of 100 reference molecules and the activity values predicted by GraphDTA. Table S6 and S7. The performance comparison between our model and baseline models on scaffold hopping generative model evaluation metrics (SEM) among five distinct targets: CDK2, JAK1, EGFR, LRRK2, and PIM1. The best 10% and 30% molecules generated by each model were evaluated. Fig. S8. The success rate of the best 30% molecules evaluated by GraphDTA and LeDock represented as a swarm plot. Swarm plot of success rate evaluated by GraphDTA for 20 reference compounds among the five targets. Fig. S9. The binding poses of the reference compound against LRRK2. The yellow dot line denotes the hydrogen bond.) [file 13321_2023_766_MOESM1_ESM.docx]

**Supporting Information for**

**ScaffoldGVAE: Scaffold Generation and Hopping of Drug Molecules**

**via a Variational Autoencoder Based on Multi-View Graph Neural**

**Networks**

Chao Hu, ^2, 3, †^ Song Li, ^1, 2, †^ Chenxing Yang, ^2^ Jun Chen, ^2^ Yi Xiong,^4, 5^ Guisheng Fan, ^3^ Hao Liu, ^2, *^ Liang Hong ^1, 4, 5 *^

^1^School of Physics and Astronomy & Institute of Natural Sciences, Shanghai Jiao Tong University, Shanghai 200240, China

^2^Shanghai Matwings Technology Co., Ltd., Shanghai, 200240, China

^3^School of Information Science and Engineering, East China University of Science and Technology, Shanghai 200237, China

^4^School of Life Sciences and Biotechnology, Shanghai Jiao Tong University, Shanghai 200240, China

^5^Zhangjiang Institute for Advanced Study, Shanghai Jiao Tong University, Shanghai 201203, China

^†^These authors contributed equally: Chao Hu, Song Li

^*^These authors jointly supervised this work: Hao Liu (chaohao2010@sjtu.edu.cn); Liang Hong ([hongl3liang@sjtu.edu.cn](mailto:hongl3liang@sjtu.edu.cn))

The molecular diagrams and scaffolds of 100 reference molecules, the red part represents the scaffold we extracted. Principle of scaffold selection: Select the scaffold that binds to the kinase hinge region as the scaffold that needs to hop.

**
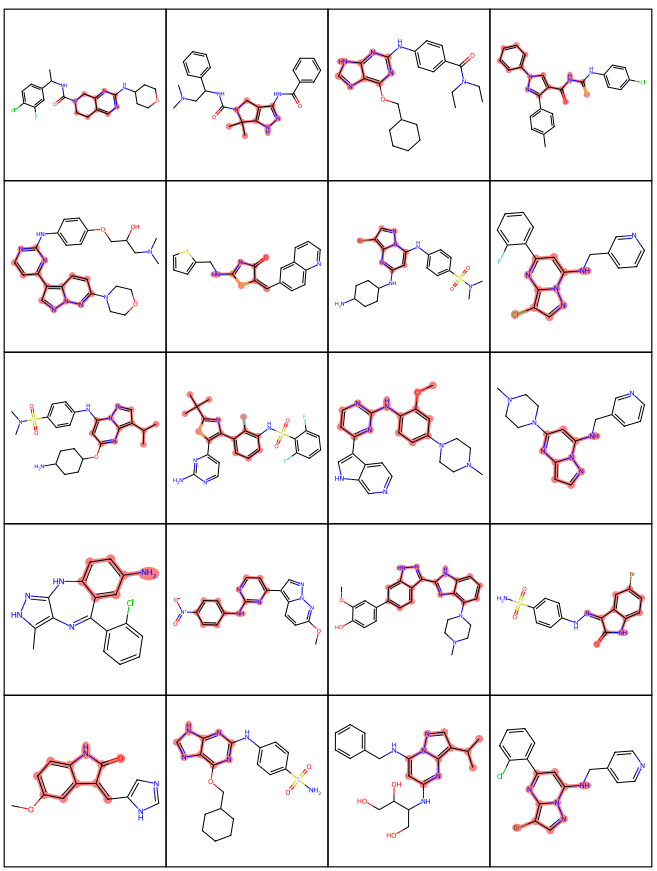
**

**Fig.S1** Reference molecules and scaffolds on the CDK2 protein

**
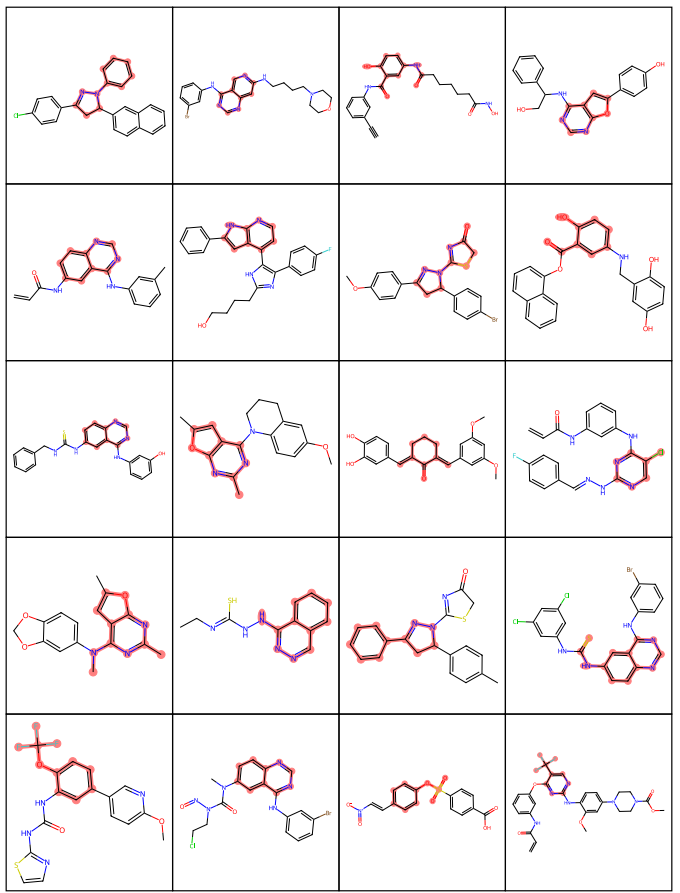
**

**Fig. S2** Reference molecules and scaffolds on the EGFR protein

**
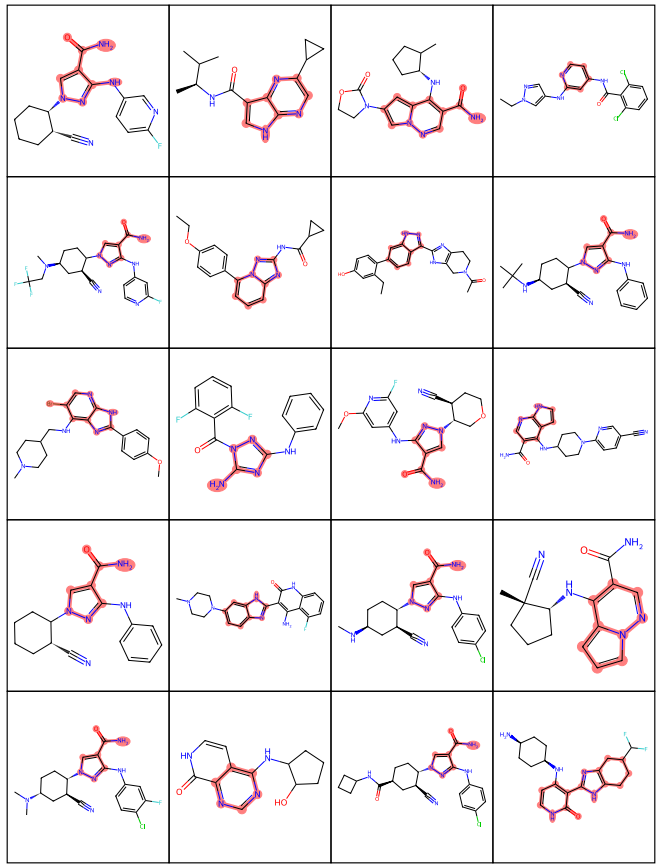
**

**Fig. S3** Reference molecules and scaffolds on the JAK1 protein

**
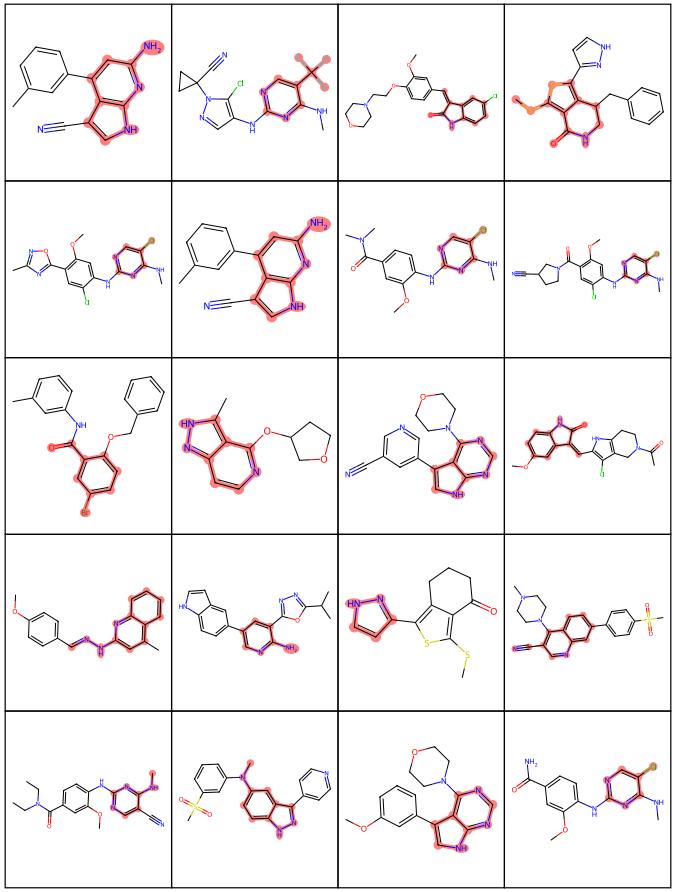
**

**Fig. S4** Reference molecules and scaffolds on the LRRK2 protein

**
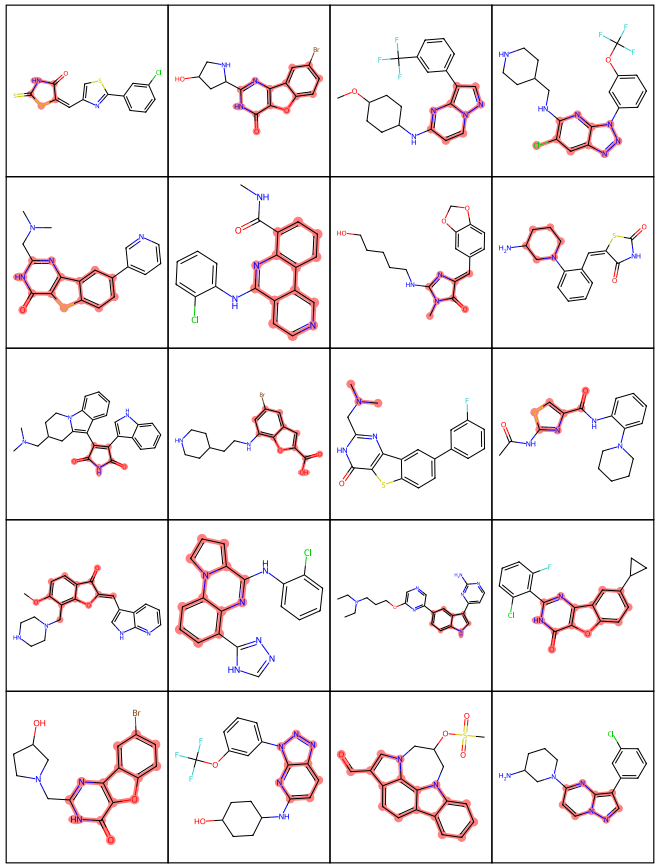
**

**Fig. S5** Reference molecules and scaffolds on the PIM1 protein

The five baselines were fine-tuned with five targets: CDK2, EGFR, JAK1, LRRK2, and PIM1, sampled, and the results of the generated molecules were calculated against the conventional molecular generation evaluation criteria.

**TableS1** The result of the AAE model on general generative model evaluation metrics (GEM)

| **Protein** | **Validity** |  | **Uniqueness1K** | **Uniqueness5K** | **Filter** | **Scaffold uniqueness** | **Scaffold novelty** | **Novelty** |
| --- | --- | --- | --- | --- | --- | --- | --- | --- |
| EGFR | 0.8802 |  | 0.8158 | 0.6842 | 0.7801 | 0.4191 | 0.2618 | 0.7593 |
| CDK2 | 0.8847 |  | 0.6757 | 0.5186 | 0.8679 | 0.3334 | 0.2174 | 0.7851 |
| JAK1 | 0.8877 |  | 0.7900 | 0.6215 | 0.9020 | 0.3495 | 0.2199 | 0.7311 |
| LRRK2 | 0.8769 |  | 0.7663 | 0.6272 | 0.9107 | 0.3677 | 0.2373 | 0.7952 |
| PIM1 | 0.8276 |  | 0.7307 | 0.5933 | 0.9135 | 0.3864 | 0.2412 | 0.7695 |

**TableS2** The result of the VAE model on general generative model evaluation metrics (GEM)

| **Protein** | **Validity** | **Uniqueness1K** | **Uniqueness5K** | **Filter** | **Scaffold uniqueness** | **Scaffold novelty** | **Novelty** |
| --- | --- | --- | --- | --- | --- | --- | --- |
| EGFR | 0.9794 | 0.8101 | 0.4848 | 0.6233 | 0.2371 | 0.1032 | 0.5090 |
| CDK2 | 0.9874 | 0.3799 | 0.1460 | 0.9177 | 0.0851 | 0.0407 | 0.5712 |
| JAK1 | 0.9773 | 0.6799 | 0.3718 | 0.9099 | 0.1792 | 0.0827 | 0.4907 |
| LRRK2 | 0.9884 | 0.5849 | 0.2426 | 0.9523 | 0.1227 | 0.0476 | 0.4396 |
| PIM1 | 0.9661 | 0.5678 | 0.2383 | 0.9211 | 0.1356 | 0.0556 | 0.4400 |

**TableS3** The result of the LatentGAN model on general generative model evaluation metrics (GEM)

| **Protein** | **Validity** | **Uniqueness1k** | **Uniqueness5K** | **Filter** | **Scaffold uniqueness** | **Scaffold novelty** | **Novelty** |
| --- | --- | --- | --- | --- | --- | --- | --- |
| EGFR | 0.7341 | 0.7307 | 0.7304 | 0.6321 | 0.7789 | 0.7160 | 1.0 |
| CDK2 | 0.6203 | 0.6172 | 0.6186 | 0.6352 | 0.8263 | 0.7807 | 1.0 |
| JAK1 | 0.5199 | 0.5237 | 0.5194 | 0.3686 | 0.9309 | 0.8989 | 1.0 |
| LRRK2 | 0.7993 | 0.7452 | 0.6998 | 0.8463 | 0.5549 | 0.5080 | 1.0 |
| PIM1 | 0.6763 | 0.6776 | 0.6673 | 0.7435 | 0.8483 | 0.8022 | 1.0 |

**TableS4** The result of the QBMG model on general generative model evaluation metrics (GEM)

| **Protein** | **Validity** | **Uniqueness1k** | **Uniqueness5K** | **Filter** | **Scaffold uniqueness** | **Scaffold novelty** | **Novelty** |
| --- | --- | --- | --- | --- | --- | --- | --- |
| EGFR | 1.0 | 1.0 | 1.0 | 0.6456 | 0.4074 | 0.2358 | 0.6942 |
| CDK2 | 1.0 | 1.0 | 1.0 | 0.8978 | 0.4903 | 0.3667 | 0.9250 |
| JAK1 | 1.0 | 1.0 | 1.0 | 0.8931 | 0.4000 | 0.2763 | 0.7803 |
| LRRK2 | 1.0 | 1.0 | 1.0 | 0.8884 | 0.4031 | 0.2758 | 0.7840 |
| PIM1 | 1.0 | 1.0 | 1,0 | 0.8543 | 0.5162 | 0.3717 | 0.8469 |

**TableS5** The result of the SyntaLinker model on general generative model evaluation metrics (GEM)

| **Protein** | **Validity** | **Uniqueness1k** | **Uniqueness5K** | **Filter** | **Scaffold uniqueness** | **Scaffold novelty** | **Novelty** |
| --- | --- | --- | --- | --- | --- | --- | --- |
| EGFR | 0.3169 | 0.3889 | 0.3145 | 0.5955 | 0.5051 | 0.4258 | 0.9964 |
| CDK2 | 0.2561 | 0.3306 | 0.2555 | 0.7791 | 0.4924 | 0.4495 | 0.9977 |
| JAK1 | 0.2204 | 0.2754 | 0.2192 | 0.8235 | 0.4962 | 0.4691 | 0.9992 |
| LRRK2 | 0.2789 | 0.3516 | 0.2784 | 0.884 | 0.4924 | 0.4473 | 0.9978 |
| PIM1 | 0.2554 | 0.3203 | 0.2544 | 0.8188 | 0.6106 | 0.5681 | 0.9984 |

**TableS6** The result of the REINVERT2 model on general generative model evaluation metrics (GEM)

| **Protein** | **Validity** | **Uniqueness1k** | **Uniqueness5K** | **Filter** | **Scaffold uniqueness** | **Scaffold novelty** | **Novelty** |
| --- | --- | --- | --- | --- | --- | --- | --- |
| EGFR | 1.0000 | 1.0000 | 1.0000 | 0.8491 | 0.9642 | 0.9452 | 1.0000 |
| CDK2 | 1.0000 | 1.0000 | 1.0000 | 0.8208 | 0.9817 | 0.9646 | 1.0000 |
| JAK1 | 1.0000 | 1.0000 | 1.0000 | 0.8642 | 0.9780 | 0.9618 | 1.0000 |
| LRRK2 | 1.0000 | 1.0000 | 1.0000 | 0.9063 | 0.9602 | 0.9357 | 0.9999 |
| PIM1 | 1.0000 | 1.0000 | 1.0000 | 0.8467 | 0.9736 | 0.9563 | 0.9999 |


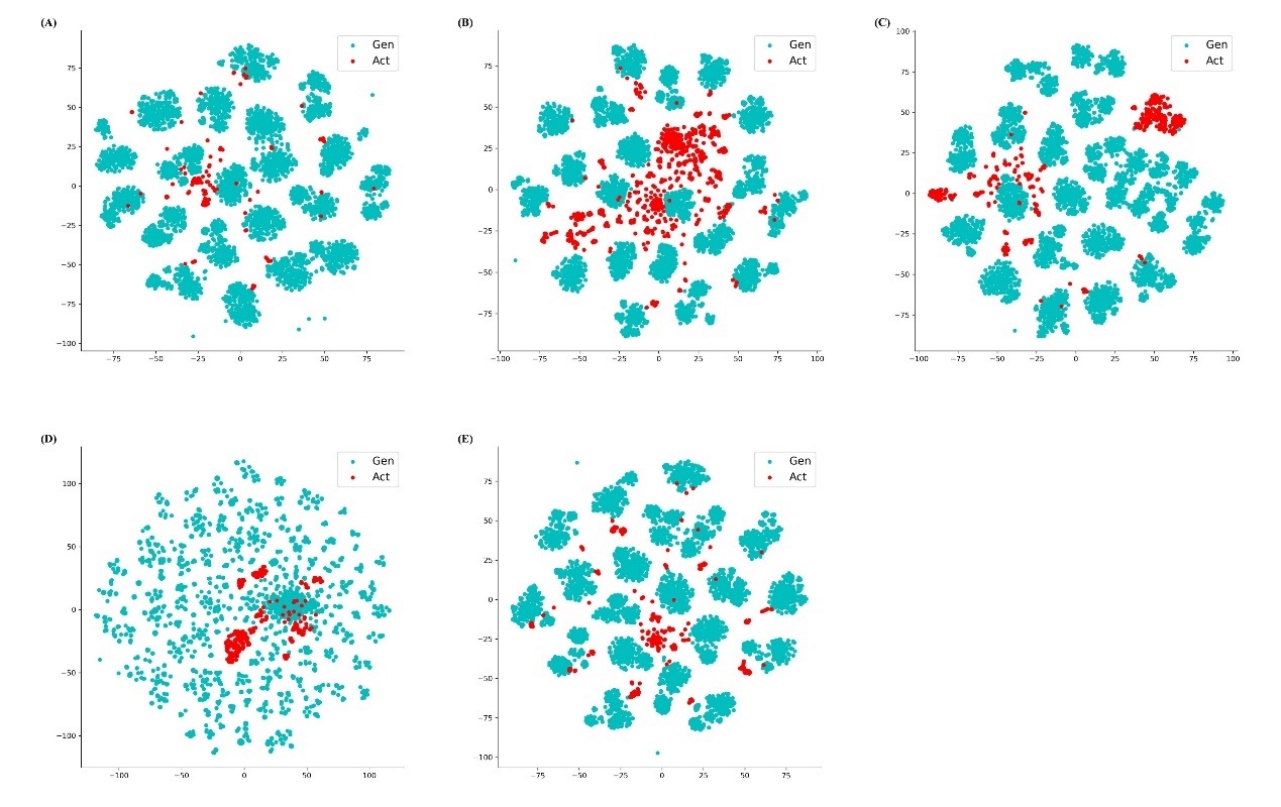


**Fig. S6** Chemical space of generated molecules and bioactive ligands of five distinct targets: (A)CDK2, (B)JAK1, (C)EGFR, (D) LRRK2, and (E)PIM1 visualized by t-SNE dimensionality reduction.

This part introduced supplementary information on scaffold hopping and activity retention evaluations. FigS6 showed the correlation between the experimental activity values of 100 reference molecules and the activity prediction of GraphDTA, to verify the accuracy of GraphDTA scores. The correlation between the two can reach 0.6590. The value of RMSE is only 0.8848. The correlation was high, indicating that GraphDTA was accurate in predicting activity. TableS6 is the top 30% of optimal results after LeDock and GraphDTA scores of 5,000 molecules generated per reference molecule. To calculate the scaffold hopping generative model evaluation metrics (SEM) and compare them with baselines. At the same time, FigS7 represents the success rate of the best 30% molecules evaluated by GraphDTA and LeDock represented as swarm plots. Swarm plot of success rate evaluated by GraphDTA for 20 reference compounds among the five targets. It is similar to the results of the first 10% of the best in the text.

**
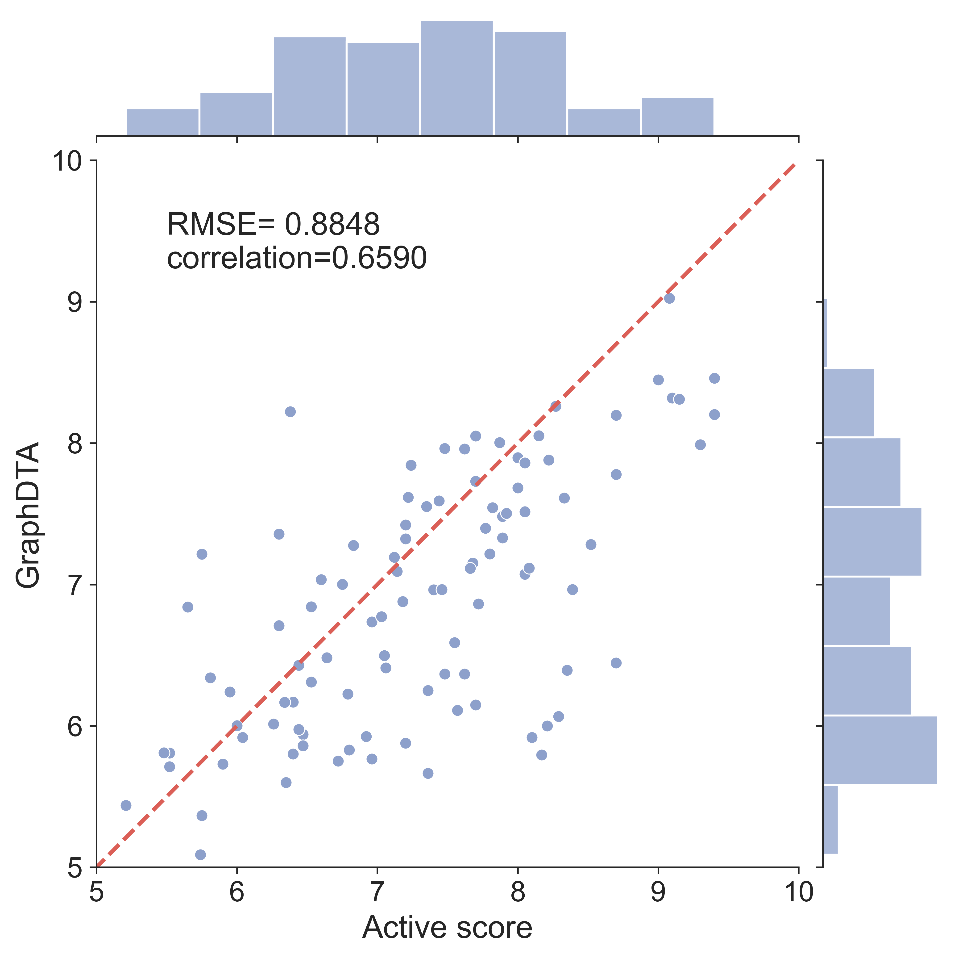
**

**Fig. S7** Correlation between the experimental activity values of 100 reference molecules and the activity values predicted by GraphDTA

**Table S6** The performance comparison between our model and baseline models on scaffold hopping generative model evaluation metrics (SEM) among five distinct targets: CDK2, JAK1, EGFR, LRRK2, and PIM1. The best 10% of molecules generated by each model were evaluated.

| Protein | Model | GraphDTA | | | | Ledock | | | | |
| --- | --- | --- | --- | --- | --- | --- | --- | --- | --- | --- |
|  |  | Active mean | Active rate | Hop rate | Success rate | Active mean | Active rate | Hop rate | Success rate | |
| CDK2 | AAE | 7.559±0.432 | 0.787±0.332 | 0.005±0.012 | 0.007±0.017 | **-9.522±0.119** | **0.950±0.122** | 0.016±0.041 | | 0.012±0.024 |
|  | VAE | **8.006±0.007** | **0.992±0.037** | 0.003±0.010 | 0.003±0.010 | -9.335±0.018 | 0.928±0.176 | 0.014±0.043 | | 0.009±0.025 |
|  | LatentGAN | 7.610±0.015 | 0.863±0.271 | 0.007±0.017 | 0.005±0.011 | -9.182±0.029 | 0.882±0.250 | 0.009±0.019 | | 0.009±0.019 |
|  | QBMG | 7.873±0.006 | 0.971±0.132 | 0.003±0.010 | 0.003±0.008 | -9.339±0.022 | 0.911±0.201 | 0.010±0.017 | | 0.008±0.014 |
|  | SyntaLinker | 6.753±0.431 | 0.469±0.423 | 0.296±0.254 | 0.113±0.160 | -8.164±0.726 | 0.559±0.381 | 0.314±0.265 | | 0.176±0.250 |
|  | REINVENT2 | 7.205±100 | 0.595±0.389 | 0.128±0.210 | 0.085±0.144 | -8.966±0.319 | 0.866±0.263 | 0.027±0.050 | | 0.027±0.050 |
|  | our | 7.151±0.361 | 0.676±0.381 | **1.000±0.000** | **0.676±0.381** | -8.208±0.863 | 0.622±0.420 | **1.000±0.000** | | **0.622±0.420** |
| EGFR | AAE | 8.295±0.134 | 0.929±0.216 | 0.007±0.017 | 0.006±0.017 | -10.880±0.036 | 0.947±0.162 | 0.003±0.007 | 0.003±0.007 | |
|  | VAE | **8.304±0.014** | **0.948± 0.160** | 0.008±0.022 | 0.008±0.022 | -10.960±0.030 | 0.945±0.170 | 0.002±0.006 | 0.002±0.006 | |
|  | LatentGAN | 7.901±0.016 | 0.872±0.287 | 0.003±0.012 | 0.003±0.012 | -10.320±0.031 | 0.913±0.270 | 0.002±0.005 | 0.002±0.005 | |
|  | QBMG | 8.221±0.008 | 0.941±0.182 | 0.006±0.017 | 0.006±0.017 | **-10.980±0.024** | **0.952±0.150** | 0.002±0.005 | 0.001±0.005 | |
|  | SyntaLinker | 6.895±0.550 | 0.506±0.348 | 0.337±0.339 | 0.167±0.256 | -8.480±0.681 | 0.721±0.396 | 0.366±0.331 | 0.331±0.347 | |
|  | REINVENT2 | 7.187±0.464 | 0.703±0.415 | 0.208±0.349 | 0.156±0.317 | -9.505±0.478 | 0.878±0.291 | 0.213±0.355 | 0.212±0.355 | |
|  | our | 7.018±0.480 | 0.613±0.439 | **1.000±0.000** | **0.613±0.439** | -8.874±1.323 | 0.923±0.241 | **1.000±0.000** | **0.923±0.241** | |
| JAK1 | AAE | 8.014±1.031 | 0.627±0.473 | 0.001±0.003 | 0.001±0.003 | **-8.979±0.205** | 0.996±0.018 | 0.001±0.002 | 0.001±0.002 | |
|  | VAE | **8.972±0.012** | **1.000±0.000** | 0.063±0.192 | 0.063±0.192 | -8.797±0.018 | 0.996±0.017 | 0.010±0.020 | 0.010±0.020 | |
|  | LatentGAN | 7.984±0.016 | 0.613±0.378 | 0.001±0.001 | 0.001±0.001 | -8.778±0.033 | 0.970±0.092 | 0.001±0.002 | 0.001±0.002 | |
|  | SyntaLinker | 7.196±0.733 | 0.283±0.238 | 0.215±0.190 | 0.053±0.079 | -7.331±0.511 | 0.498±0.303 | 0.233±0.184 | 0.109±0.125 | |
|  | QBMG | 8.920±0.008 | **1.000±0.000** | 0.053±0.164 | 0.053±0.164 | -8.957±0.020 | **1.000±0.000** | 0.012±0.025 | 0.012±0.025 | |
|  | REINVENT2 | 6.862±0.217 | **0.300**±0.421 | 0.077±0.201 | 0.062±0.199 | -8.504±0.374 | 0.871±0.247 | 0.001±0.004 | 0.001±0.004 | |
|  | our | 7.739±0.370 | 0.510±0.444 | **0.952± 0.213** | **0.462±0.440** | -7.861±0.619 | 0.721±0.369 | **0.955±0.200** | **0.676±0.387** | |
| LRRK2 | AAE | 7.109±0.119 | **1.000±0.000** | 0.062±0.145 | 0.062±0.145 | -8.471±0.571 | **0.992±0.037** | 0.064±0.177 | 0.064±0.177 | |
|  | VAE | 7.177±0.014 | **1.000±0.000** | 0.033±0.054 | 0.033±0.054 | -7.888±0.016 | 0.991±0.039 | 0.065±0.169 | 0.065±0.161 | |
|  | LatentGAN | 6.981±0.014 | 0.988±0.053 | 0.042±0.076 | 0.042±0.076 | -7.878±0.019 | 0.986±0.061 | 0.072±0.149 | 0.072±0.149 | |
|  | SyntaLinker | 6.542±0.332 | 0.860±0.219 | 0.355±0.273 | 0.295±0.274 | -6.980±0.649 | 0.839±0.308 | 0.373±0.286 | 0.336±0.288 | |
|  | QBMG | **7.485±0.090** | **1.000±0.000** | 0.072±0.163 | 0.072±0.163 | **-8.562±0.486** | 0.950±0.223 | 0.028±0.048 | 0.022±0.042 | |
|  | REINVENT2 | 6.836±0.212 | 0.973±0.120 | 0.245**±0.325** | 0.244**±0.326** | -8.400±0.355 | 1.000±0.000 | 0.242±0.315 | 0.242±0.315 | |
|  | our | 6.663±0.267 | 0.924±0.190 | **1.000±0.000** | **0.924±0.190** | -7.205±0.781 | 0.928±0.229 | **1.000±0.000** | **0.928±0.229** | |
| PIM1 | AAE | 8.179±0.251 | 0.893±0.272 | 0.007±0.027 | 0.007±0.027 | **-7.869±0.394** | 0.988±0.052 | 0.005±0.019 | 0.005±0.019 | |
|  | VAE | **9.012±0.035** | **0.971±0.128** | 0.013±0.036 | 0.011±0.034 | -7.582±0.020 | 0.986±0.043 | 0.016±0.040 | 0.015±0.041 | |
|  | LatentGAN | 8.059±0.015 | 0.885±0.273 | 0.016±0.064 | 0.015±0.061 | -7.454±0.013 | 0.956±0.137 | 0.014±0.058 | 0.014±0.058 | |
|  | SyntaLinker | 7.329±0.528 | 0.622±0.378 | 0.239±0.275 | 0.129±0.169 | -6.626±0.750 | 0.645±0.377 | 0.242±0.263 | 0.142±0.229 | |
|  | QBMG | 8.648±0.018 | 0.949±0.179 | 0.013±0.037 | 0.012±0.037 | -7.661±0.016 | **0.991±0.030** | 0.012±0.033 | 0.012±0.033 | |
|  | REINVENT2 | 7.930±0.406 | 0.834±0.319 | 0.193±0.316 | 0.154±0.254 | -7.699±0.357 | **0.992**±0.034 | 0.172±0.303 | 0.172±0.303 | |
|  | our | 8.310±0.259 | 0.943±0.216 | **1.000±0.000** | **0.943±0.216** | -6.879±0.697 | 0.796±0.347 | **1.000±0.000** | **0.796±0.347** | |

**Table S7** the performance comparison between our model and baseline models on scaffold hopping generative

model evaluation metrics (SEM) among five distinct targets: CDK2, JAK1, EGFR, LRRK2, and PIM1. The best

30% of molecules generated by each model were evaluated.

| Protein | Model | GraphDTA | | | | Ledock | | | |
| --- | --- | --- | --- | --- | --- | --- | --- | --- | --- |
|  |  | Active mean | Active rate | Hop rate | Success rate | Active mean | Active rate | Hop rate | Success rate |
| CDK2 | AAE | 7.169±0.537 | 0.654±0.409 | 0.012±0.018 | 0.005±0.008 | **-8.963±0.067** | **0.819±0.302** | 0.013±0.023 | 0.010±0.016 |
|  | VAE | **7.795±0.010** | **0.936±0.173** | 0.007±0.014 | 0.003±0.006 | -8.903±0.015 | 0.817±0.315 | 0.012±0.020 | 0.008±0.012 |
|  | LatentGAN | 7.152±0.015 | 0.587±0.351 | 0.009±0.020 | 0.003±0.006 | -8.599±0.018 | 0.710±0.355 | 0.010±0.023 | 0.010±0.023 |
|  | QBMG | 7.533±0.009 | 0.809±0.268 | 0.005±0.007 | 0.003±0.004 | -8.778±0.023 | 0.772±0.334 | 0.007±0.010 | 0.006±0.009 |
|  | SyntaLinker | 6.202±0.413 | 0.270±0.285 | 0.306±0.240 | 0.080±0.150 | -7.494±0.808 | 0.347±0.298 | 0.309±0.239 | 0.108±0.180 |
|  | REINVENT2 | 6.789±0.123 | 0.428±0.427 | 0.131±0.218 | 0.066±0.126 | -8.285±0.309 | 0.625±0.349 | 0.027±0.059 | 0.025±0.059 |
|  | our | 6.853±0.434 | 0.499±0.433 | **1.000±0.000** | **0.499±0.433** | -7.787±0.884 | 0.441±0.401 | **1.000±0.000** | **0.441±0.401** |
| EGFR | AAE | 7.895±0.135 | 0.838±0.305 | 0.005±0.010 | 0.004±0.009 | -9.965±0.083 | 0.888±0.266 | 0.004±0.008 | 0.004±0.008 |
|  | VAE | **7.941±0.010** | **0.872±0.273** | 0.005±0.013 | 0.005±0.013 | -10.070±0.023 | 0.892±0.264 | 0.003±0.008 | 0.003±0.008 |
|  | LatentGAN | 7.505±0.017 | 0.737±0.366 | 0.003±0.010 | 0.003±0.009 | -9.596±0.027 | 0.856±0.309 | 0.003±0.007 | 0.003±0.007 |
|  | QBMG | 7.864±0.007 | 0.853±0.291 | 0.005±0.011 | 0.004±0.010 | **-10.120±0.020** | **0.903±0.254** | 0.003±0.008 | 0.003±0.008 |
|  | SyntaLinker | 6.340±0.478 | 0.291±0.268 | 0.344±0.337 | 0.087±0.139 | -7.851±0.641 | 0.484±0.340 | 0.349±0.338 | 0.251±0.302 |
|  | REINVENT2 | 6.771±0.473 | 0.529±0.420 | 0.209±0.347 | 0.104±0.244 | -8.865±0.476 | 0.778±0.360 | 0.213±0/354 | 0.213±0.354 |
|  | our | 6.663±0.517 | 0.443±0.414 | **1.000±0.000** | **0.443±0.414** | -8.365±1.353 | 0.731±0.317 | **1.000±0.000** | **0.731±0.317** |
| JAK1 | AAE | 7.468±1.285 | 0.545±0.473 | 0.003±0.012 | 0.003±0.012 | **-8.442±0.175** | 0.892±0.215 | 0.003±0.009 | 0.001±0.004 |
|  | VAE | **8.685±0.008** | **0.989±0.034** | 0.056±0.148 | 0.054±0.145 | -8.351±0.014 | **0.902±0.216** | 0.025±0.056 | 0.024±0.056 |
|  | LatentGAN | 7.316±0.024 | 0.396±0.389 | 0.001±0.002 | 0.001±0.001 | -8.091±0.026 | 0.780±0.275 | 0.001±0.001 | 0.001±0.001 |
|  | SyntaLinker | 6.600±0.665 | 0.174±0.194 | 0.229±0.172 | 0.030±0.044 | -6.789±0.526 | 0.288±0.229 | 0.232±0.175 | 0.055±0.053 |
|  | QBMG | 8.655±0.006 | 0.981±0.059 | 0.046±0.136 | 0.046±0.135 | -8.405±0.015 | 0.903±0.207 | 0.019±0.049 | 0.019±0.049 |
|  | REINVENT2 | 6.226±0.236 | 0.130±0.205 | 0.075±0.201 | 0.034±0.125 | -7.849±0.428 | 0.726±0.357 | 0.002±0.006 | 0.002±0.006 |
|  | our | 7.415±0.467 | 0.366±0.418 | **0.952±0.216** | **0.331±0.417** | -7.360±0.627 | 0.511±0.399 | **0.954±0.204** | **0.465±0.392** |
| LRRK2 | AAE | **6.809±0.145** | 0.948±0.111 | 0.050±0.121 | 0.049±0.120 | **-8.006±0.435** | **0.964±0.161** | 0.057±0.149 | 0.057±0.149 |
|  | VAE | 6.802±0.013 | 0.950±0.146 | 0.050±0.091 | 0.048±0.091 | -7.602±0.011 | 0.964±0.162 | 0.086±0.187 | 0.086±0.187 |
|  | LatentGAN | 6.630±0.012 | 0.882±0.213 | 0.054±0.096 | 0.051±0.096 | -7.511±0.012 | 0.948±0.175 | 0.088±0.171 | 0.083±0.162 |
|  | SyntaLinker | 6.173±0.255 | 0.590±0.246 | 0.373±0.279 | 0.227±0.247 | -6.401±0.580 | 0.563±0.302 | 0.375±0.276 | 0.247±0.220 |
|  | QBMG | 7.282±0.123 | **1.000±0.000** | 0.056±0.115 | 0.056±0.115 | -7.961±0.344 | 0.933±0.232 | 0.036±0.062 | 0.029±0.058 |
|  | REINVENT2 | 6.549±0.200 | 0.891±0.212 | 0.243±0.324 | 0.239±0.324 | -7.823±0.382 | 0.962±0.112 | 0.235±0.311 | 0.235±0.3111 |
|  | our | 6.401±0.247 | 0.776±0.333 | **1.000±0.000** | **0.776±0.333** | -6.741±0.804 | 0.720±0.326 | **1.000±0.000** | **0.720±0.326** |
| PIM1 | AAE | 7.683±0.263 | 0.715±0.347 | 0.006±0.016 | 0.004±0.012 | **-7.339±0.357** | **0.926±0.189** | 0.005±0.016 | 0.004±0.016 |
|  | VAE | **8.273±0.017** | **0.887±0.243** | 0.015±0.031 | 0.013±0.028 | -7.172±0.014 | 0.916±0.220 | 0.013±0.031 | 0.012±0.031 |
|  | LatentGAN | 7.658±0.012 | 0.713±0.353 | 0.013±0.052 | 0.006±0.021 | -6.93±0.012 | 0.854±0.270 | 0.013±0.052 | 0.013±0.052 |
|  | SyntaLinker | 6.586±0.444 | 0.367±0.269 | 0.233±0.26 | 0.068±0.074 | -6.215±0.664 | 0.458±0.339 | 0.232±0.253 | 0.083±0.123 |
|  | QBMG | 8.071±0.011 | 0.849±0.282 | 0.012±0.028 | 0.009±0.022 | -7.182±0.011 | 0.910±0.217 | 0.010±0.026 | 0.010±0.026 |
|  | REINVENT2 | 7.499±0.417 | 0.644±0.383 | 0.188±0.311 | 0.096±0.221 | -7.168±0.337 | 0.917±0.209 | 0.175±0.304 | 0.175±0.304 |
|  | our | 7.898±0.329 | 0.854±0.292 | **1.000±0.000** | **0.854±0.292** | -6.435±0.707 | 0.606±0.385 | **1.000±0.000** | **0.606±0.385** |


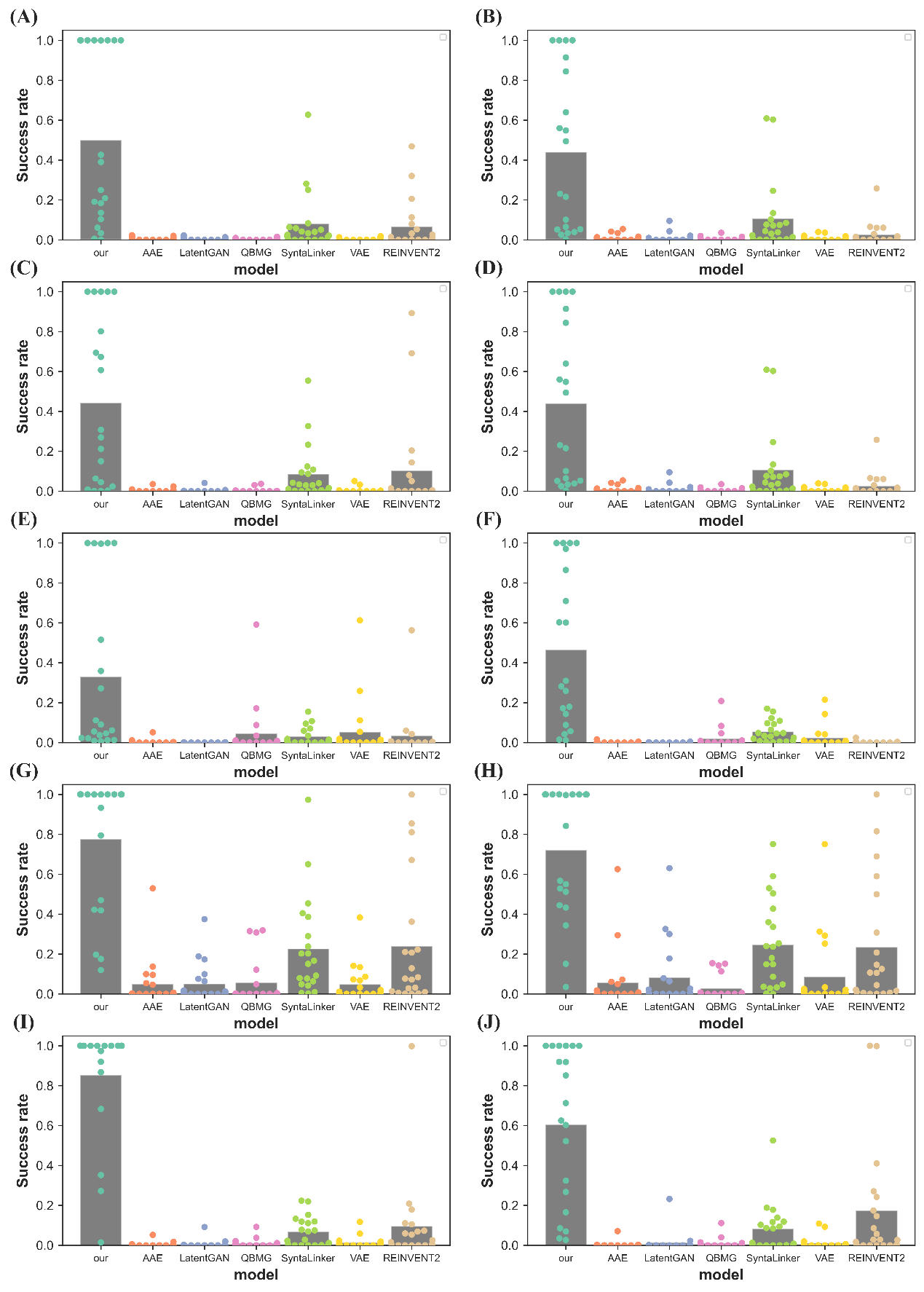


**Fig. S8** The success rate of the best 30% molecules evaluated by GraphDTA and LeDock represented as a swarm plot. Swarm plot of success rate evaluated by GraphDTA for 20 reference compounds among the target of (A) CDK2; (C) EGFR; (E) JAK1; (G) LRRK2; (I) PIM1. Swarm plot of success rate evaluated by LeDock for 20 reference compounds among the target of (B) CDK2; (D) EGFR; (F) JAK1; (H) LRRK2; (J) PIM1. The points denote the success rate of generated molecules, and the gray bars are the average success rate for 20 reference compounds.

**
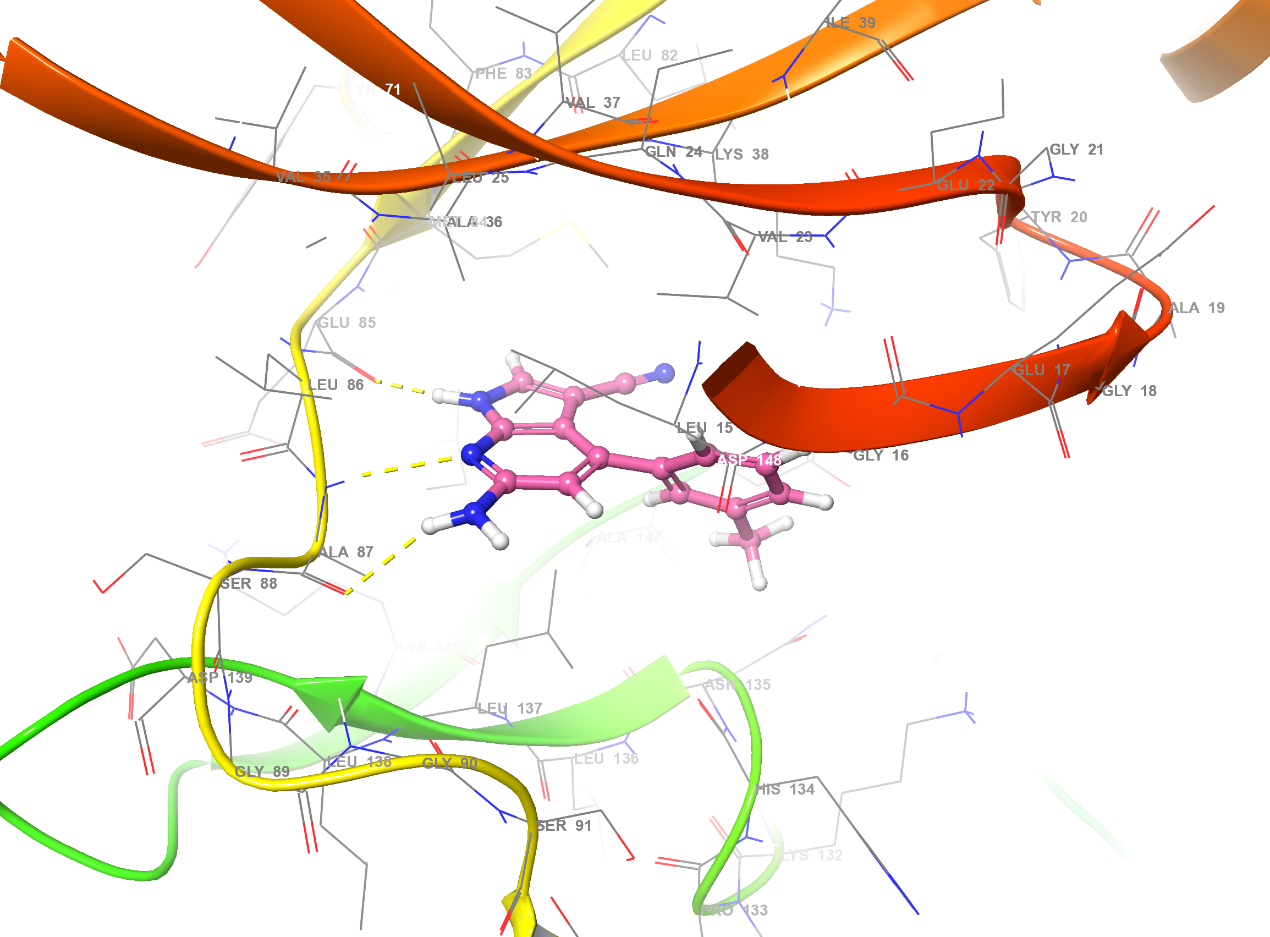
**

**Fig. S9** The binding poses of the reference compound against LRRK2. the yellow dot line denotes the hydrogen bond.
